# Proteomics-based insights into mitogen-activated protein kinase inhibitor resistance of cerebral melanoma metastases

**DOI:** 10.1186/s12014-018-9189-x

**Published:** 2018-03-09

**Authors:** Nina Zila, Andrea Bileck, Besnik Muqaku, Lukas Janker, Ossia M. Eichhoff, Phil F. Cheng, Reinhard Dummer, Mitchell P. Levesque, Christopher Gerner, Verena Paulitschke

**Affiliations:** 1 Department of Dermatology, Medical University of Vienna, Waehringer Guertel 18–20, 1090 Vienna, Austria; 2 Department of Analytical Chemistry, Faculty of Chemistry, University of Vienna, Vienna, Austria; 3 University of Applied Sciences (FH Campus Wien), Vienna, Austria; 4 Department of Dermatology, University of Zurich, University Hospital Zurich, Zurich, Switzerland

**Keywords:** BRAF mutation, Cerebral melanoma metastases, Drug resistance, Melanoma, MAP kinase inhibitor, Proteomics

## Abstract

**Background:**

MAP kinase inhibitor (MAPKi) therapy for BRAF mutated melanoma is characterized by high response rates but development of drug resistance within a median progression-free survival (PFS) of 9–12 months. Understanding mechanisms of resistance and identifying effective therapeutic alternatives is one of the most important scientific challenges in melanoma. Using proteomics, we want to specifically gain insight into the pathophysiological process of cerebral metastases.

**Methods:**

Cerebral metastases from melanoma patients were initially analyzed by a LC–MS shotgun approach performed on a QExactive HF hybrid quadrupole-orbitrap mass spectrometer. For further validation steps after bioinformatics analysis, a targeted LC-QQQ-MS approach, as well as Western blot, immunohistochemistry and immunocytochemistry was performed.

**Results:**

In this pilot study, we were able to identify 5977 proteins by LC–MS analysis (data are available via ProteomeXchange with identifier PXD007592). Based on PFS, samples were classified into good responders (PFS ≥ 6 months) and poor responders (PFS $\le$ 3 months). By evaluating these proteomic profiles according to gene ontology (GO) terms, KEGG pathways and gene set enrichment analysis (GSEA), we could characterize differences between the two distinct groups. We detected an EMT feature (up-regulation of N-cadherin) as classifier between the two groups, V-type proton ATPases, cell adhesion proteins and several transporter and exchanger proteins to be significantly up-regulated in poor responding patients, whereas good responders showed an immune activation, among other features. We identified class-discriminating proteins based on nearest shrunken centroids, validated and quantified this signature by a targeted approach and could correlate parts of this signature with resistance using the CPL/MUW proteome database and survival of patients by TCGA analysis. We further validated an EMT-like signature as a major discriminator between good and poor responders on primary melanoma cells derived from cerebral metastases. Higher immune activity is demonstrated in patients with good response to MAPKi by immunohistochemical staining of biopsy samples of cerebral melanoma metastases.

**Conclusions:**

Employing proteomic analysis, we confirmed known extra-cerebral resistance mechanisms in the cerebral metastases and further discovered possible brain specific mechanisms of drug efflux, which might serve as treatment targets or as predictive markers for these kinds of metastasis.

**Electronic supplementary material:**

The online version of this article (10.1186/s12014-018-9189-x) contains supplementary material, which is available to authorized users.

## Background

The incidence of melanoma is increasing rapidly worldwide, including countries with historically low rates. This increase is occurring at a faster rate than with any other neoplasm [1]. Up to one-fifth of melanoma patients develop metastatic disease, which has mainly been treated by chemotherapy, achieving response rates between 10 and 30% [2]. After cancers of the lung and breast, melanoma is the third most common cause of central nervous system (CNS) metastases [3, 4]. In patients with newly diagnosed stage IV disease, brain metastases are present in 20% of cases [5] and among patients with documented brain involvement, these lesions contribute to death in up to 95% [6]. In 40–60% of all melanoma patients, mutational activation of BRAF V600E can be found, resulting in constitutive activation of the serine-threonine protein kinase BRAF and the Ras-RAF-MEK-ERK signaling pathway, also known as mitogen activated protein (MAP) kinase pathway [7]. Until 2011, standard treatment for patients with inoperable metastatic melanoma was dacarbazine (DTIC) [8]. In August 2011, the US Agency of Food and Drug Administration (FDA) approved Vemurafenib, a BRAF inhibitor (BRAFi), for the treatment of metastatic or unresectable melanoma with BRAF V600E mutation. Brain metastases pose special challenges because of the poor associated prognosis [9]. They are a major cause of mortality in patients with advanced melanoma. However, relatively little was known about the intracranial effectiveness of selective inhibitors because patients with brain metastases have historically been excluded from clinical trials [10]. Recently a few studies investigated BRAFi treatment for melanoma patients with brain metastasis [11–13]. An open-label pilot study assessed Vemurafenib therapy in patients with BRAF V600 mutation positive metastatic melanoma with non-resectable, previously treated brain metastases and concluded that Vemurafenib can safely be used for the therapy of advanced symptomatic melanoma with metastasis to the brain and can result in meaningful tumor regression [11]. In more than 50% of these cases clinical response, up to a complete remission, were achieved by Vemurafenib. Systemic therapy revolutionized melanoma treatment but unfortunately high initial responses are followed by acquired drug resistance after a median time of only 6–8 months [14, 15]. About 15% of the patients treated with BRAFi do not achieve tumor regression, because of intrinsic (primary) mechanisms of resistance and most patients who respond to therapy ultimately develop mechanisms of acquired (secondary) resistance, leading to progressive disease [16]. Initial success was made by BRAFi therapy, but due to the reactivation of the MAPK signaling pathway, patients showed resistance to therapy. Therefore a combination therapy of BRAF and MEK inhibitors (MEKi) was implemented and improved the effectiveness of targeted therapy even further by improving median PFS [17, 18]. Unfortunately, resistance to therapy is still challenging and especially the high mortality of brain metastases poses a huge ongoing clinical problem. It is questionable whether there are brain specific efflux transporters contributing to the resistance of intracranial metastases and where differences or similarities between visceral and cerebral metastases can be found. The phenotype-switching model was first described in melanoma by Hoek et al. [19, 20] and characterizes two transcriptional distinct melanoma cell populations, a proliferative and an invasive type. Melanoma cells are able to switch back and forth between these two phenotypes and therefore explain the heterogeneous nature of melanoma cells [21]. It accounts for disease progression and tumor heterogeneity, as well as aspects of resistance [22]. Proliferative melanoma cells have been shown to be more responsive to MAPK pathway inhibition than invasive phenotype cells, independently of their mutation status [23, 24]. On the other hand, proliferative melanoma cells have shown to change their phenotype from proliferative to invasive state in response to long-term treatment with targeted therapy (BRAFi and MEKi), which is associated with drug resistance. The major role in phenotype switching and the transition from a proliferative to an invasive cell type and ultimately leading to metastasis is played by EMT-like mechanisms [25]. Identifying critical switches in EMT processes and finding a way to block these, might serve as a strategy to prevent metastasis and to decrease therapeutic resistance. Especially, the down-regulation of E-cadherin, balanced by the increased expression of mesenchymal neural cadherin (N-cadherin), this, so called cadherin switch, alters cell adhesion [26, 27] and is considered to be a fundamental event in EMT, leading to a loss of cell–cell contact, increased invasive properties and typical morphological changes [21, 28, 29].

In this study we showed, by analyzing brain tissue samples of patients with low and high PFS in response to MAPKi treatment, distinct differences between the two groups, using proteome profiling by shotgun MS. We analyzed the data according to gene ontology (GO) terms, KEGG pathways and gene set enrichment analysis (GSEA). We were further able to identify an EMT mechanism (up-regulation of N-cadherin), V-type proton ATPases, calcium ion binding proteins, eukaryotic translation initiation factors, cell adhesion proteins, several transporter and exchanger proteins in poor responding patients, whereas good responders showed an immune activation and involvement of extracellular matrix structural constituents, among other features. Based on nearest shrunken centroids we furthermore detected the most discriminating proteins between those two groups. We validated the EMT like signature using primary melanoma cells derived from cerebral metastases with different response based on the IC50 to BRAF and MEK inhibitors. To validate the discriminative signature we used the CPL/MUW proteome database [30]. By a subsequent targeted approach, we performed a quantification and validation step of this discriminative signature. Functional analysis of the EMT signature was performed by inducing EMT in a primary melanoma cell culture. As a final step we validated our findings clinically by correlating our protein signature with survival over TCGA analysis and immunohistochemical staining (for study design see Additional file 1: Fig. S1).

## Methods

All experiments were carried out according to the Declaration of Helsinki principles after approval by the ethics committee (Swissethics, Kantonale Ethikkommission Zurich, vote number 2014-0425). Cerebral melanoma metastases (n = 25) originating from surgical excisions and autopsy specimens were selected based on the clinical information and patients’ different progression-free survival (PFS) after MAPKi treatment. For subsequent data analysis patients with a PFS ≥ 6 months were classified as a good responder (n = 9) and showed low progression, whereas patients with a PFS ≤ 3 months were classified as a poor responder (n = 16) and showed fast progression in their disease. Further validation steps included melanoma cells derived from cerebral metastases (n = 5) and FFPE (formalin-fixed paraffin-embedded) cerebral melanoma metastases tissue samples (n = 23), corresponding to patients from the initial metastases cohort. A clinical table with age, gender, treatment, response distribution, PFS, mutational status, all number of samples, IC50 of the cell systems and the applied methods can be found as Additional file 2: Table S1.

### LC–MS sample preparation

2–5 mg of each cryopreserved tissue sample and cell pellets from confluent 75 cm^2^ tissue culture flasks were homogenized in 100 μl sample buffer (7.5 M Urea, 1.5 M Thiourea, 0.1 M dithiothreitol (DTT), 4% 3-[(3-Cholamidopropyl)dimethylammonio]-1-propanesulfonate (CHAPS), 0.05% Sodium dodecyl sulfate (SDS)) using ultrasound. Protein concentrations were determined according to Bradford et al. [31]. For proteome analysis we prepared in-solution digests using a variation of the FASP protocol [32], as previously described [33]. Of each lysate 20 µg protein was concentrated onto a 10 kDa MWCO filter (molecular weight cut-off filter; Pall Nanosep Centrifugal Devices with Omega Membrane, #OD010), which was prewashed with LC–MS grade water (Merck Chemicals and Life Science GesmbH, Vienna, Austria), by centrifugation at 14,000×*g* for 15 min to remove all particles smaller than 10 kDa. Samples containing proteins were then reduced with 200 µl dithiothreitol (DTT) solution (5 mg/ml dissolved in 8 M guanidinium hydrochloride in 50 mM ammonium bicarbonate buffer, pH 8) and incubated at 56 °C for 30 min. After centrifugation at 14,000×*g* for 10 min, a washing step with 50 mM ammonium bicarbonate buffer was performed. For alkylation 200 µl iodoacetamide (IAA) solution (10 mg/ml in 8 M guanidinium hydrochloride in 50 mM ammonium bicarbonate buffer) was added and incubated at 30 °C for 30 min in the dark. After centrifugation at 14,000×*g* for 10 min, proteins on top of the filters were washed with 50 mM ammonium bicarbonate buffer. Afterwards, filters were placed in a new Eppendorf tube, and 100 µl of 50 mM ammonium bicarbonate buffer as well as 10 µl of protease solution (Promega Trypsin/Lys-C Mix, Mass Spec Grade, #V5073, 0.1 µg/µl) were added, and incubated at 37 °C for 18 h. After digestion, peptide samples were cleaned up with C-18 spin columns (Thermo Fisher Scientific Pierce C18 spin columns, #89870). Peptides were collected with 0.5% trifluoroacetic acid (TFA) and acidified to a final concentration of 1% TFA. C-18 columns were prewashed two times with 500 µl acetonitrile (ACN) and equilibrated with 200 µl of 5% ACN and 0.5% TFA by centrifugation at 1500×*g* for 1 min. Eluted and acidified peptide samples were loaded onto prewashed and equilibrated spin columns. After centrifugation at 1500×*g* for 1 min, the flow-through was reloaded on the column to maximize peptide binding and again centrifuged. After a washing step with 5% ACN and 0.5% TFA, peptides were eluted twice with 40 µl 50% ACN and 0.1% TFA and once with 40 µl 80% ACN and 0.1% TFA into a new Eppendorf tube. Digested peptide samples were finally dried at 40 °C using a centrifugal vacuum concentrator (miVac GeneVac Duo Concentrator) and stored at − 20 °C until further MS analyses were performed.

### LC–MS/MS shotgun analysis

As described previously [33, 34], dried samples were reconstituted in 5 µl 30% formic acid (FA) containing 10 fmol each of 4 synthetic standard peptides (allowing us to monitor the quality of the chromatographic separation) and diluted with 40 µl mobile phase A (98% H_2_O, 2% ACN, 0.1% FA). Of this solution 2.5 µl were injected into the Dionex Ultimate 3000 nano HPLC-system (Thermo Fisher Scientific). Peptides were first concentrated on a 2 cm × 75 µm C18 Pepmap100 pre-column (Thermo Fisher Scientific) at a flow rate of 10 µl/min using mobile phase A. Afterwards, separation of the peptides was achieved by eluting them from the pre-column to a 50 cm × 75 µm Pepap100 analytical column (Thermo Fisher Scientific) applying a flow rate of 300 nl/min and using a gradient of 8% to 40% mobile phase B (80% ACN, 20% H_2_O, 0.1% FA), over 190 min for the analysis of samples. The mass spectrometric analysis, with a technical replicate for each of the 18 samples, was performed on a QExactive HF orbitrap mass spectrometer, equipped with a nanospray ion source (Thermo Fisher Scientific), coupled to the nano HPLC system. For detection, MS scans were performed in the range from m/z 400–1400 at a resolution of 60,000 (at m/z = 200). MS/MS scans were performed choosing a top 12 method; HCD fragmentation was applied at 27% normalized collision energy and analysis in the orbitrap at a resolution of 15,000 (at m/z = 200).

### LC–MS shotgun data analysis

Protein inference as well as label-free quantitative (LFQ) data analysis was performed using the open source software MaxQuant 1.3.0.5 including the Andromeda search engine and the Perseus statistical analysis package [35, 36], a commonly used workflow for processing and statistical assessment of shotgun proteomics data. Protein inference was achieved searching against *homo sapiens* in the SwissProt Database (version 01/2013 with 20,264 entries) allowing a mass tolerance of 5 ppm for MS spectra and 20 ppm for MS/MS spectra as well as a maximum of 2 missed cleavages. In addition, carbamidomethylation on cysteins was included as fixed modification whereas methionine oxidation as well as N-terminal protein acetylation was included as variable modifications. Furthermore, search criteria included a minimum of two peptide identifications per protein, at least one of them unique, and the FDR calculation based on q-values performed for both, peptide identification as well as protein inference, less than 0.01. Prior to statistical analysis, proteins were filtered for reversed sequences, contaminants and a minimum of three independent identifications per protein. The mass spectrometry-based proteomics data have been deposited to the ProteomeXchange Consortium via the PRIDE [37] partner repository with the dataset identifier PXD007592 and https://doi.org/10.6019/pxd007592. Label-free quantification resulted in LFQ values for each individual protein and was used for quantitative assessment of protein regulation. For all samples the same initial protein amount of 20 µg was used and served for normalization. Using the Perseus statistical analysis package, differences of LFQ values were calculated. By applying a two-sided *t* test with *p* < 0.05 and an FDR-based permutation correction, significantly up- and down-regulated proteins with a minimum of a twofold abundance difference (log_2_ fold change) were determined. All proteins meeting these criteria were considered in the present study. Subsequent annotation enrichment analysis was performed based on gene ontology terms biological process, cellular component and molecular function using DAVID (DAVID Bioinformatics Resources 6.7, National Institute of Allergy and Infectious Diseases) [38, 39]. Kyoto Encyclopedia of Genes and Genomes (KEGG) pathways according to Geiger et al. [40] were visualized using Pathview package of R [41]. The expression of proteins was also analyzed for significantly enriched protein sets using Gene Set Enrichment Analysis (GSEA) [42]. Process using the GSEA software with the parameters set to ranking according to log fold change, minimum gene set size 15, maximum gene set size 500. To identify a potential biomarker profile, a classifier based on nearest shrunken centroids was constructed using ClaNC and R [43]. Priors were chosen according to the number of samples. The performance of the classifier was tested by leaving-one-out cross validation.

### Targeted LC–MS analysis

MRM method was developed based on shotgun data and using Skyline software (v.4.1) [44], as described recently [45]. Targeted MRM analysis was conducted on an Agilent 6490 triple quadrupole mass spectrometer coupled with a nano-Chip-LC Agilent Infinity Series HPLC1290 system. Peptides were separated by applying 19 min gradient from 8 to 30% acetonitrile. The statistical analysis of MRM data was performed with MSstats (v.2.3.5) [46].

### Cell culture

Melanoma cell cultures were established from surplus cerebral melanoma metastases after having obtained written, informed consent approved by the local IRB (EK647 and EK800). Cells were grown in RPMI (Sigma RPMI-1640, #R0883) supplemented with 5 mM l-glutamine (gibco l-glutamine, #25030), 1 mM sodium pyruvate (Sigma sodium pyruvate, #S8636) and 10% FBS (PAN biotech FBS Premium heat inactivated, #P30-1902, Aidenbach, Germany). Culture medium was changed every 2–3 days to ensure optimum conditions of growth, using aseptic techniques and a laminar flow bench. Cells were tested for mycoplasma contamination (Invivogen PlasmoTest Mycoplasma Detection Kit, #rep-pt) prior to their use for the described techniques.

### Viability assay

Melanoma cells were seeded into 96-well plates in a density of 1.5 × 10^3^ cells per well. After 24 h cells were treated with Raf inhibitors Encorafenib (Selleckchem LGX818, #S7108) or Vemurafenib (Selleckchem PLX4032, RG7204, #S1267) and MEK inhibitor Binimetinib (Selleckchem MEK162, ARRY-162, ARRY-438162, #S7007) in different concentrations and incubated for 72 h. Proliferation and viability of the cells was determined with a standard colorimetric assay using 7-hydroxy-3H-phenoxazin-3-one-10-oxide sodium salt (Sigma Resazurin sodium salt, #R7017), where the bioreduction of the dye was measured at 595 nm. IC50 values were determined by nonlinear regression using the dose–response equations built into GraphPad Prism software version 5 (GraphPad Software Inc., USA).

### Western blot

Patient derived melanoma cells were washed twice with cold PBS (gibco PBS, pH 7.4 (1X) without calcium or magnesium, #10010) and lysed with RIPA cell lysis buffer (20 mM Tris–HCl, pH 7.5, 150 mM NaCl, 5 mM EDTA 1% Triton-X 100, 1 mM Na_3_VO_4_, 1X protease inhibitor cocktail (Roche cOmplete ULTRA Tablets, Mini, *EASYpack*, #05 892 970 001), 1X phosphatase inhibitor cocktail (Roche PhosSTOP *EASYpack*, #05 906 837 001)). Total protein content was quantified using a colorimetric assay (Bio-Rad DC Protein Assay, #5000112) according to the manufacturer’s protocol with bovine serum albumin (BSA) as standard. SDS–polyacrylamide gels were hand casted with 8% separating gel and for each sample 5 µg of total protein was loaded and separated by electrophoresis for 150 min by applying 100 V (using Bio-Rad Mini-Protean Tetra Cell). Using a semi-dry transfer unit (Hoefer TE70X semi-dry blotter) proteins were blotted onto a nitrocellulose membrane (Bio-Rad Nitrocellulose membrane 0.45 µm, #162-0115) for 90 min at 70 mA. After a blocking step for 1 h at room temperature with 5% milk (Bio-Rad nonfat dry milk Blotting-Grade Blocker, #1706404) in TBS-T under agitation, the primary antibody (Cell Signaling E-Cadherin (24E10) Rabbit mAb, #3195; Cell Signaling N-Cadherin (D4R1H) XP Rabbit mAb, #13116) diluted 1:1000 in TBS-T was incubated overnight at 4 °C. After a washing step, the secondary antibody (Amersham ECL Peroxidase labelled anti-rabbit antibody, #NA934) was diluted 1:2000 in 5% milk in TBS-T and incubated under agitation for 1 h at room temperature. After a washing step bound antibodies were detected with enhanced chemiluminescence horseradish peroxidase (Thermo Fisher Scientific SuperSignal West Pico Chemiluminescent Substrate, #34080) in the dark room.

### Immunohistochemical staining

Cerebral melanoma metastases tissue used for immunohistochemistry was fixed in 4% formaldehyde solution and embedded in paraffin. The 4 µm sections were then deparaffinized in xylene and rehydrated. Epitope retrieval was performed in antibody specific buffers (Agilent Dako Target Retrieval Solution, pH9). Staining was performed on an immunohistochemistry stainer (Agilent Dako Autostainer Link 48) using a labeled streptavidin–biotin method visualized by AEC as chromogen (Agilent Dako REAL Detection System, Peroxidase/AEC, Rabbit/Mouse, #K5003). The antibodies used were: CD3 (Dako Monoclonal Mouse Anti-Human CD3 Clone F7.2.38, #M7254; 1:50); CD4 (Dako Monoclonal Mouse Anti-Human CD4 Clone 4B12, #M7310; 1:80); CD8 (Biocare Medical CD8 [C8/144B], #ACI3160A; 1:300). All slides were counterstained with hematoxylin (Agilent Dako REAL Hematoxylin, #S2020).

### Immunocytochemical staining

Melanoma cell lines from cerebral metastases were taken out of cell culture and fixated with 7.5% formaldehyde solution. After two washing steps with PBS cells were concentrated onto glass slides using a cytospin centrifuge. Immunocytochemical staining was performed using a biotin-free detection system with horseradish peroxidase polymer and AEC chromogen (Thermo Fisher Scientific Lab Vision UltraVision LP Detection System, #TL-015-HA). The antibodies used (Cell Signaling E-Cadherin (24E10) Rabbit mAb, #3195; Cell Signaling N-Cadherin (D4R1H) XP Rabbit mAb, #13116) were diluted 1:1000 in 1% BSA in PBS and incubated overnight at 4 °C. After chromogenic development all slides were counterstained with hematoxylin (Merck Papanicolaou’s solution 1a Harris’ hematoxylin, #109253).

### TGFβ treatment, RNA extraction and microarray data analysis

The BRAFV600E mutated primary human melanoma cell line M000921 has been established from surplus material from cutaneous melanoma metastases. This cell line has been previously characterized as a proliferative-phenotype melanoma culture (by means of melanoma phenotype-switching model) and shared with multiple studies and international laboratories [24, 47–50]. Expression data from a Affymetrix HG-U133 plus 2.0 oligonucleotide microarray is deposited on GEO (GSM700745). Written informed consent was approved by the local IRB (EK647 and EK800). Clinical diagnosis was confirmed by histology and immunohistochemistry. Melanoma cell culture was grown in RPMI (Sigma RPMI-1640, #R0883) including 5 mM l-glutamine (gibco l-glutamine, #25030), 1 mM sodium pyruvate (Sigma sodium pyruvate, #S8636) and 10% FBS (PAN biotech FBS Premium heat inactivated, #P30-1902, Aidenbach, Germany). M000921 melanoma cells were kept in medium containing 5 ng/ml human recombinant TGFβ (R&D Systems, #240-B) for a period of 12 days. Medium containing fresh TGFβ protein was changed every 3 days. RNA was extracted from both untreated and TGFβ treated cell culture using TRIzol reagent (Invitrogen, USA) following manufacturer’s protocol. RNA labelling, hybridization to microarray (HG-U133 plus 2.0, Affymetrix) and data were statistically analyzed as described previously [49].

### TCGA data

Gene expression and clinical information were derived from The Cancer Genome Atlas (TCGA, cutaneous melanoma dataset (n = 456)) [51]. Briefly, patients were segregated according to the upper and lower quartile of gene expression and significant differences in survival rates are evaluated with the log-rank statistical test (*p* value < 0.05). The survival curves were visualized by Kaplan–meier plots.

## Results

### Proteome profiling of cerebral melanoma metastases

In this study we generated proteome profiling data out of cerebral melanoma metastases by shotgun proteomics. As a result, a total of 5977 proteins were detected in the tissue samples, assembled from 49,501 distinct peptides, were identified and assessed using a label-free quantification approach. Like previously described, the shotgun proteomic strategy has a unique potential to discover novel functional aspects of proteins and to determine relative abundance levels of proteins identified in different samples [52]. For our comparison we assigned the samples to two groups based on patients’ PFS. For further evaluation, all proteins with *p* < 0.05 and fold change differences $\ge$ 2.0 (student t-test difference) were taken into consideration. This corresponded to 1636 proteins more abundant in the poor responder group (patients with PFS $\le$ 3 months) and 271 proteins in the good responder group (patients with PFS $\ge$ 6 months).

### Functional discrimination of the subgroups

**Fig. 1:**
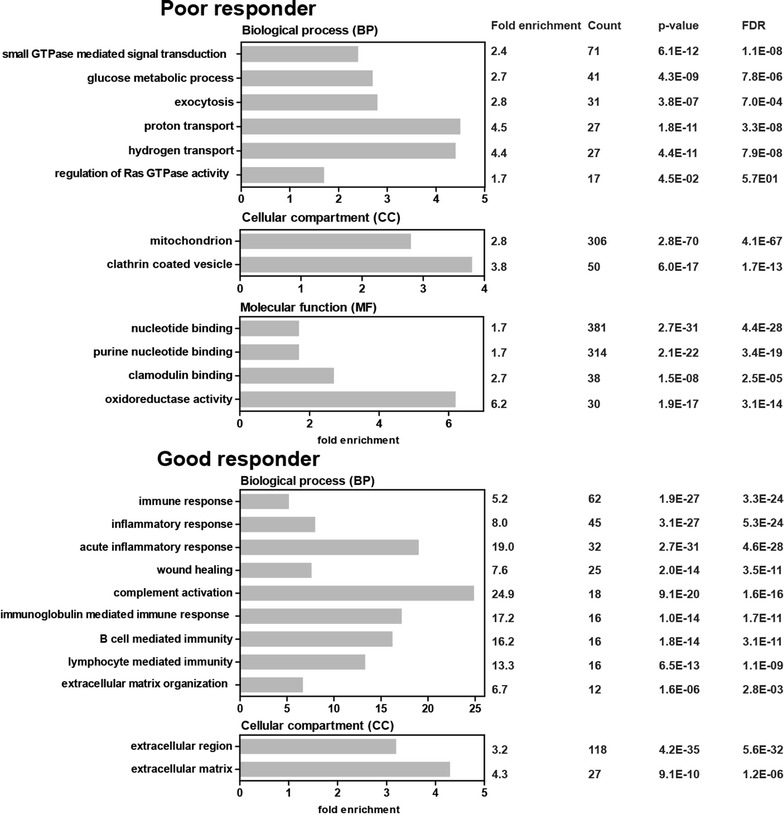
Gene annotation enrichment analysis using the concept of GO annotations for poor responder (left column) and good responder (right column). Classification by the GO term biological process (BP) shows pathways and larger processes made up of the activities of multiple gene products, classification by the GO term cellular component (CC) shows where gene products are active and classification by the GO term molecular function (MF) shows molecular activities of gene products. Fold enrichment values for individual GO terms, count (genes involved in the term), *p* value and FDR (false discovery rate, calculated using the Benjamini–Hochberg procedure), listed next to the graph, were calculated using DAVID bioinformatics resources

**Fig. 2:**
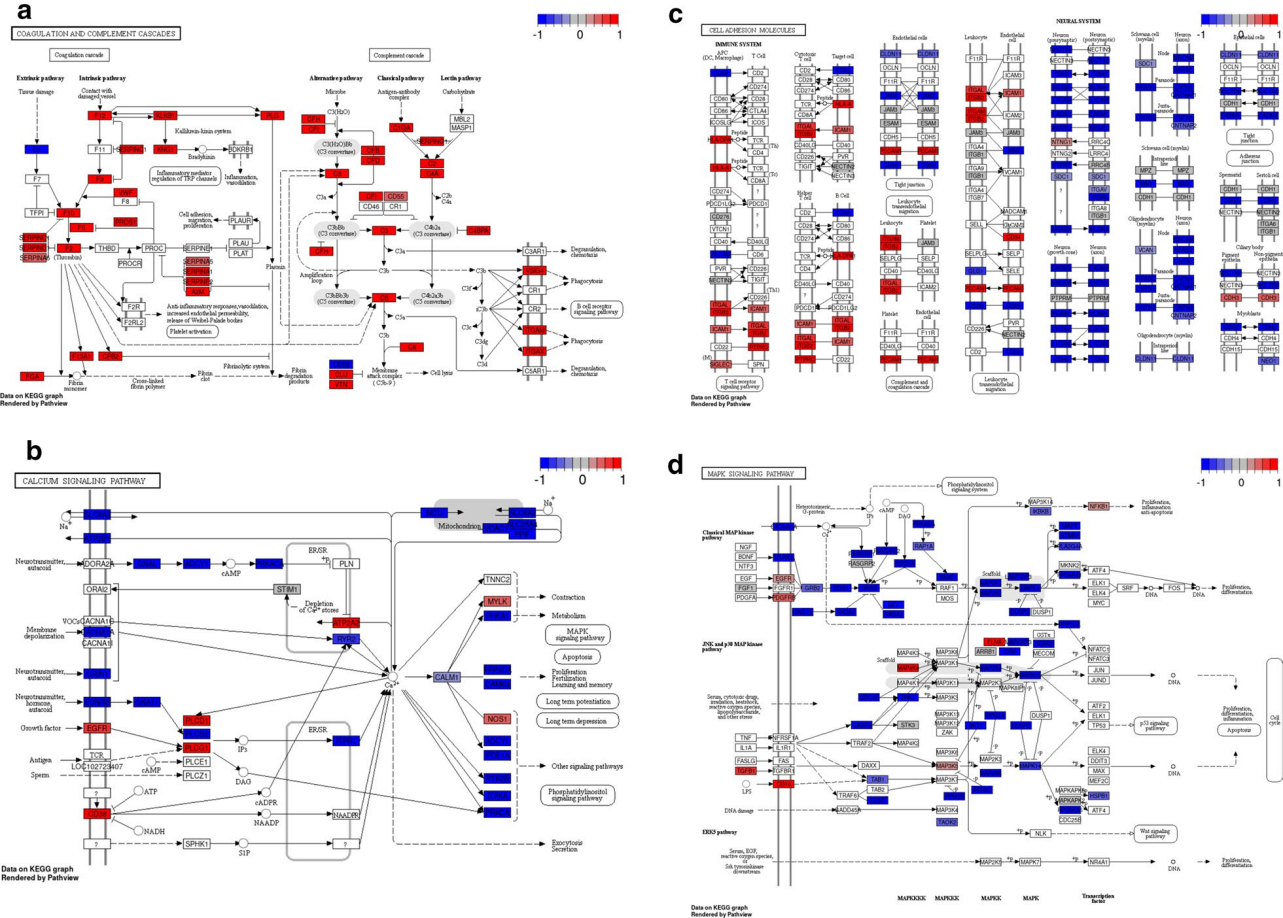
KEGG pathway visualization of the coagulation and complement cascades (**a**), cell adhesion molecules (**b**), calcium signaling pathway (**c**) and MAPK signaling pathway (**d**). Red: up-regulated in good responder; blue: down-regulated in good responder

For a functional discrimination between good and poor responder we used gene ontology (GO) annotations (Fig. 1). In order to gain a better understanding on the molecular interaction and connection of the individual candidates we mapped differentially regulated proteins to functional KEGG (Kyoto Encyclopedia of Genes and Genomes) pathways (Fig. 2). Furthermore, Gene Set Enrichment Analysis (GSEA) [42] was applied and showed very similar results (see Additional file 3: Fig. S2 for enriched pathways and Additional file 4: Table S2 for GSEA statistics). By both methods, we could show that cell adhesion molecules, calcium signaling pathway and MAPK signaling pathway proteins were overrepresented in poor responders, while complement and coagulation cascade proteins were overrepresented in good responders.

**Fig. 3:**
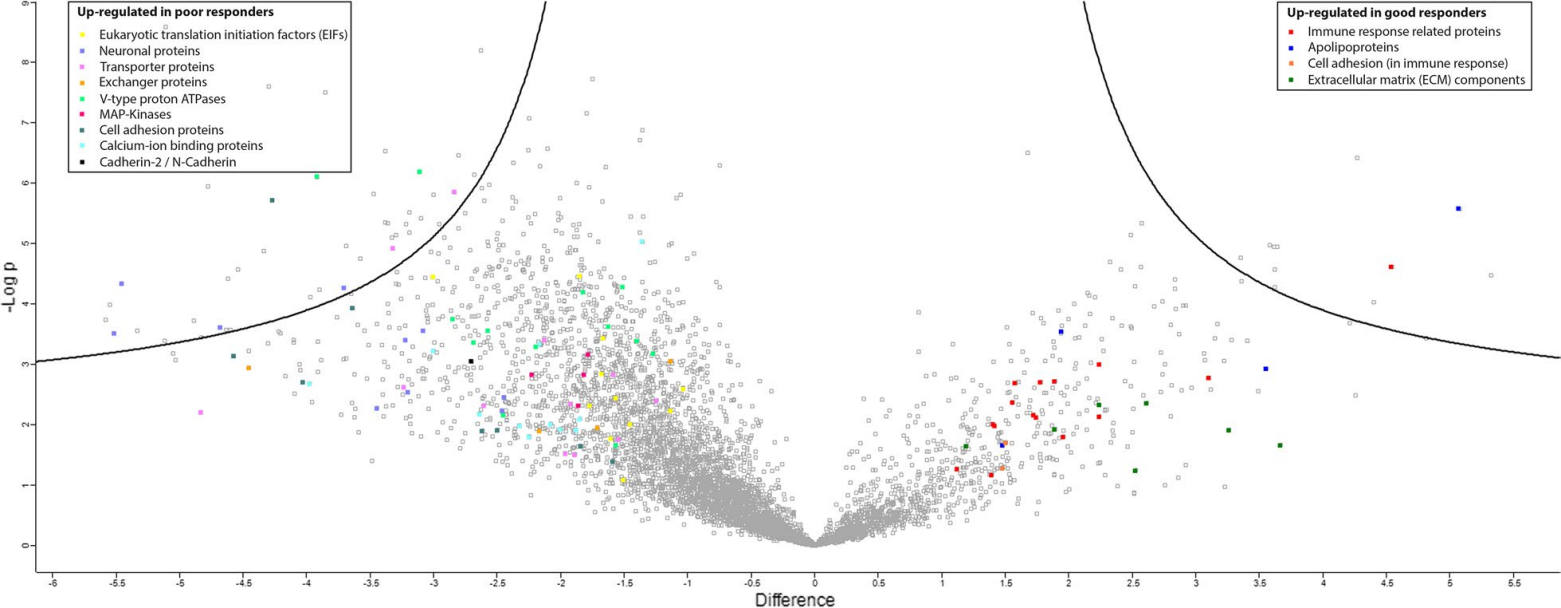
Regulation of proteins in patients with poor and good response. The volcano plot shows the difference in the LFQ values (fold change, logarithmic scale to the base of two) on the x-axis including their corresponding *p* values (logarithmic scale) on the y-axis. Extended information on the proteins can be found in Tables 1 and 2

We identified significant differentially expressed protein groups between the good (with higher PFS) and poor (with lower PFS), shown in the volcano plot (Fig. 3).

Identification of proteins involved in the MAPK signaling pathway, V-type proton ATPases, calcium ion binding proteins, eukaryotic translation initiation factors, proteins involved in cell adhesion, neuronal proteins, transporter and exchanger proteins were significantly up-regulated in the poor responder group compared to the good responder group. Samples from patients who responded poorly to MAPKi treatment also showed a significant higher expression of Cadherin-2 (CDH2), also known as N-Cadherin.

Whereas patients who responded well to MAPKi showed a strong immunogenic signature, as well as proteins associated with the extracellular matrix (ECM), a subgroup of cell adhesion proteins, which are involved in immunological responses and apolipoproteins were found to be elevated amongst this subgroup. Individual proteins can be found in Tables 1 and 2.

**Table 1:** Proteins significantly up-regulated in poor responder patients as indicated in the volcano plot

Acc. Nr.	Protein name	Gene name	log_2_ fold change (t-test difference)	*p* value	t-test significant
*Eukaryotic translation initiation factors (EIFs)*					
Q14232	Translation initiation factor eIF-2B subunit alpha	EIF2B1	1.04	2.48E − 03	
Q14152	Eukaryotic translation initiation factor 3 subunit A	EIF3A	1.13	5.71E − 03	
P55884	Eukaryotic translation initiation factor 3 subunit B	EIF3B	1.57	3.62E − 03	
O00303	Eukaryotic translation initiation factor 3 subunit F	EIF3F	1.23	9.41E − 03	
O75821	Eukaryotic translation initiation factor 3 subunit G	EIF3G	1.59	4.81E − 02	
Q13347	Eukaryotic translation initiation factor 3 subunit I	EIF3I	2.88	4.29E − 05	*
Q9UBQ5	Eukaryotic translation initiation factor 3 subunit K	EIF3 K	1.85	3.50E − 05	
Q9Y262	Eukaryotic translation initiation factor 3 subunit L	EIF3L	1.57	7.30E − 03	
P23588	Eukaryotic translation initiation factor 4B	EIF4B	1.69	1.44E − 03	
Q15056	Eukaryotic translation initiation factor 4H	EIF4H	1.68	3.92E − 04	
O60841	Eukaryotic translation initiation factor 5B	EIF5B	1.26	2.53E − 02	
*Neuronal proteins*					
Q8N111	Cell cycle exit and neuronal differentiation protein 1	CEND1	2.86	1.98E − 03	
P51674	Neuronal membrane glycoprotein M6-a	GPM6A	5.30	5.24E − 05	*
P62166	Neuronal calcium sensor 1	NCS1	3.55	6.65E − 05	*
Q7Z3B1	Neuronal growth regulator 1	NEGR1	3.71	1.93E − 03	
Q15818	Neuronal pentraxin-1	NPTX1	3.07	4.79E − 03	
Q92823	Neuronal cell adhesion molecule	NRCAM	2.44	3.48E − 03	
Q9UH03	Neuronal-specific septin-3	SEPT3	3.40	3.68E − 04	
P17600	Synapsin-1	SYN1	4.68	2.47E − 04	*
Q92777	Synapsin-2	SYN2	5.49	3.17E − 04	*
O14994	Synapsin-3	SYN3	2.46	5.70E − 03	
*Transporter proteins*					
P30531	Sodium- and chloride-dependent GABA transporter 1	SLC6A1	3.44	8.85E − 06	*
P43003	Excitatory amino acid transporter 1	SLC1A3	3.17	2.51E − 03	
P43004	Excitatory amino acid transporter 2	SLC1A2	4.98	5.16E − 03	
P43007	Neutral amino acid transporter A	SLC1A4	1.81	4.10E − 02	
P48066	Sodium- and chloride-dependent GABA transporter 3	SLC6A11	2.12	4.95E − 04	
Q15043	Zinc transporter ZIP14	SLC39A14	1.28	3.30E − 03	
Q6PML9	Zinc transporter 9	SLC30A9	1.57	1.36E − 02	
Q8N4V1	Membrane magnesium transporter 1	MMGT1	1.19	3.22E − 02	
Q8TBB6	Probable cationic amino acid transporter	SLC7A14	1.78	6.86E − 03	
Q96QE2	Proton myo-inositol cotransporter	SLC2A13	2.68	1.94E − 06	*
Q99726	Zinc transporter 3	SLC30A3	2.03	2.95E − 02	
Q9H1V8	Sodium-dependent neutral amino acid transporter SLC6A17	SLC6A17	2.51	1.39E − 02	
Q9NS82	Asc-type amino acid transporter 1	SLC7A10	1.44	6.39E − 03	
*Exchanger proteins*					
P32418	Sodium/calcium exchanger 1	SLC8A1	1.66	4.06E − 02	
Q8TCU6	Phosphatidylinositol 3,4,5-trisphosphate-dependent Rac exchanger 1 protein	PREX1	1.03	1.03E − 03	
Q92581	Sodium/hydrogen exchanger 6	SLC9A6	1.76	9.73E − 03	
Q9UPR5	Sodium/calcium exchanger 2	SLC8A2	4.39	1.18E − 03	
*V-type proton ATPases*					
Q15904	V-type proton ATPase subunit S1	ATP6AP1	1.41	3.12E − 02	
Q93050	V-type proton ATPase 116 kDa subunit a isoform 1	ATP6V0A1	2.57	2.75E − 04	
P27449	V-type proton ATPase 16 kDa proteolipid subunit	ATP6V0C	2.36	5.37E − 03	
P61421	V-type proton ATPase subunit d 1	ATP6V0D1	3.96	8.92E − 07	*
P38606	V-type proton ATPase catalytic subunit A	ATP6V1A	1.82	6.37E − 05	
P21281	V-type proton ATPase subunit B, brain isoform	ATP6V1B2	1.62	2.37E − 04	
P21283	V-type proton ATPase subunit C 1	ATP6V1C1	1.51	5.27E − 05	
Q9Y5K8	V-type proton ATPase subunit D	ATP6V1D	2.19	5.15E − 04	
P36543	V-type proton ATPase subunit E 1	ATP6V1E1	1.27	6.60E − 04	
Q16864	V-type proton ATPase subunit F	ATP6V1F	3.12	5.47E − 07	*
O75348	V-type proton ATPase subunit G 1	ATP6V1G1	2.39	5.16E − 04	
O95670	V-type proton ATPase subunit G 2	ATP6V1G2	1.40	4.11E − 04	
Q9UI12	V-type proton ATPase subunit H	ATP6V1H	2.75	4.83E − 04	
*MAP-kinases*					
P28482	Mitogen-activated protein kinase 1	MAPK1	1.78	6.70E − 04	
P53779	Mitogen-activated protein kinase 10	MAPK10	2.27	1.27E − 03	
P27361	Mitogen-activated protein kinase 3	MAPK3	1.79	1.39E − 03	
Q9UPT6	C-Jun-amino-terminal kinase-interacting protein 3	MAPK8IP3	2.21	1.83E − 03	
*Cell adhesion proteins*					
O00533	Neural cell adhesion molecule L1-like protein	CHL1	1.59	3.85E − 02	
O15394	Neural cell adhesion molecule 2	NCAM2	2.56	1.84E − 02	
P13591	Neural cell adhesion molecule 1	NCAM1	3.72	1.12E − 04	*
P32004	Neural cell adhesion molecule L1	L1CAM	4.27	1.91E − 06	*
Q14982	Opioid-binding protein/cell adhesion molecule	OPCML	4.53	7.99E − 04	*
Q14CZ8	Hepatocyte cell adhesion molecule	HEPACAM	2.49	1.23E − 02	
Q8N3J6	Cell adhesion molecule 2	CADM2	4.32	1.24E − 03	
Q8NFZ8	Cell adhesion molecule 4	CADM4	1.74	2.53E − 02	
Q92823	Neuronal cell adhesion molecule	NRCAM	2.44	3.48E − 03	
*Calcium-ion binding proteins*					
P62158	Calmodulin	CALM1	1.36	9.22E − 06	
Q14012	Calcium/calmodulin-dependent protein kinase type 1	CAMK1	2.17	4.56E − 04	
Q9UQM7	Calcium/calmodulin-dependent protein kinase type II subunit alpha	CAMK2A	3.00	5.85E − 04	
Q13554	Calcium/calmodulin-dependent protein kinase type II subunit beta	CAMK2B	3.98	2.08E − 03	
Q13555	Calcium/calmodulin-dependent protein kinase type II subunit gamma	CAMK2G	2.77	5.24E − 03	
Q16566	Calcium/calmodulin-dependent protein kinase type IV	CAMK4	1.98	1.25E − 02	
Q8N5S9	Calcium/calmodulin-dependent protein kinase kinase 1	CAMKK1	2.13	8.09E − 03	
Q96RR4	Calcium/calmodulin-dependent protein kinase kinase 2	CAMKK2	2.03	8.67E − 03	
Q9P1Y5	Calmodulin-regulated spectrin-associated protein 3	CAMSAP3	2.35	8.13E − 03	
P54750	Calcium/calmodulin-dependent 3,5-cyclic nucleotide phosphodiesterase 1A	PDE1A	1.97	5.00E − 03	
Q01064	Calcium/calmodulin-dependent 3,5-cyclic nucleotide phosphodiesterase 1B	PDE1B	2.54	1.25E − 02	
*EMT*					
P19022	Cadherin-2	CDH2	2.53	1.02E − 03	

The table shows UniProt accession number (Acc. Nr.), protein name, gene name, t-test difference between LFQ values of poor and good responding patients (fold change, logarithmic scale to the base of two) with corresponding *p* values and t-test significance (rows with a t-test result above s0 = 0.5 and FDR 0.01 are reported as significant)

**Table 2:** Proteins significantly up-regulated in good responder patients as indicated in the volcano plot

Acc. Nr.	Protein name	Gene name	log_2_ fold change (t-test difference)	*p* value	t-test significant
*Immune response related proteins*					
P05164	Myeloperoxidase	MPO	4.53	1.69E − 05	*
P01909	HLA class II histocompatibility antigen, DQ alpha 1 chain	HLA-DQA1	1.43	1.01E − 03	
P04233	HLA class II histocompatibility antigen gamma chain	CD74	1.74	7.47E − 03	
P20039	HLA class II histocompatibility antigen, DRB1-11 beta chain	HLA-DRB1	2.99	2.46E − 03	
Q95365	HLA class I histocompatibility antigen, B-38 alpha chain	HLA-B	1.84	3.64E − 03	
Q5Y7A7	HLA class II histocompatibility antigen, DRB1-13 beta chain	HLA-DRB1	1.38	1.70E − 03	
P01594	Ig kappa chain V-I region AU		1.07	7.46E − 03	
P01609	Ig kappa chain V-I region Scw		1.58	2.02E − 03	
P01613	Ig kappa chain V-I region Ni		1.86	1.68E − 02	
P01617	Ig kappa chain V-II region TEW		1.61	4.60E − 03	
P01623	Ig kappa chain V-III region WOL		1.89	1.87E − 03	
P01777	Ig heavy chain V-III region TEI		1.78	1.97E − 03	
P01767	Ig heavy chain V-III region BUT		1.46	3.39E − 02	
P01779	Ig heavy chain V-III region TUR		2.24	9.82E − 04	
P01834	Ig kappa chain C region	IGKC	1.56	4.17E − 03	
P01857	Ig gamma-1 chain C region	IGHG1	1.40	9.49E − 03	
P01860	Ig gamma-3 chain C region	IGHG3	1.42	1.01E − 02	
P01871	Ig mu chain C region	IGHM	2.24	7.25E − 03	
P01876	Ig alpha-1 chain C region	IGHA1	1.12	5.26E − 02	
P01880	Ig delta chain C region	IGHD	1.36	4.04E − 02	
*Apolipoproteins*					
O14791	Apolipoprotein L1	APOL1	1.82	1.43E − 03	
P02647	Apolipoprotein A-I	APOA1	1.94	2.86E − 04	
P04114	Apolipoprotein B-100	APOB	3.55	1.17E − 03	
P06727	Apolipoprotein A-IV	APOA4	5.06	2.61E − 06	*
*Cell adhesion (in immune response)*					
P16284	Platelet endothelial cell adhesion molecule	PECAM1	1.48	5.04E − 02	
P31997	Carcinoembryonic antigen-related cell adhesion molecule 8	CEACAM8	1.86	3.08E − 03	
P40199	Carcinoembryonic antigen-related cell adhesion molecule 6	CEACAM6	1.16	3.99E − 02	
*Extracellular matrix (ECM) components*					
P02452	Collagen alpha-1(I) chain	COL1A1	3.67	2.18E − 02	
P02751	Fibronectin	FN1	2.61	4.26E − 03	
P08123	Collagen alpha-2(I) chain	COL1A2	3.24	1.25E − 02	
P25067	Collagen alpha-2(VIII) chain	COL8A2	1.29	9.84E − 03	
P39059	Collagen alpha-1(XV) chain	COL15A1	1.48	4.46E − 02	
Q05707	Collagen alpha-1(XIV) chain	COL14A1	2.53	5.55E − 02	
Q96P44	Collagen alpha-1(XXI) chain	COL21A1	1.78	3.42E − 02	

The table shows UniProt accession number (Acc. Nr.), protein name, gene name, t-test difference between LFQ values of poor and good responding patients (fold change, logarithmic scale to the base of two) with corresponding *p* values and t-test significance (rows with a t-test result above s0 = 0.5 and FDR 0.01 are reported as significant)

### Identification of discriminative proteins

**Fig. 4:**
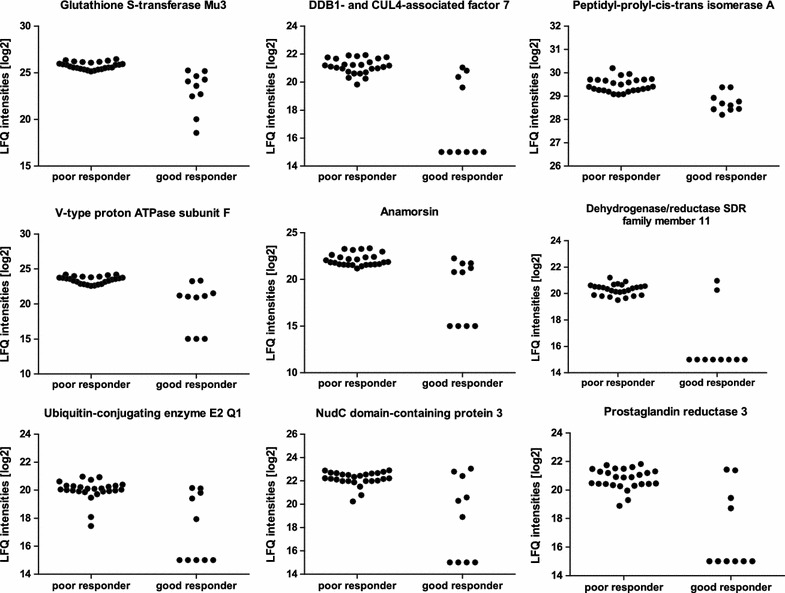
Protein panel displays differences between poor and good responders. For each of the 9 most class-discriminating proteins (listed in Table 3), label-free quantification (LFQ) intensities in a logarithmic scale to the basis 2 are indicated. LFQ intensities for proteins not detected in a replicate were replaced by 15

To generalize our proteomic findings and enable the translation of the results towards clinical application, we constructed a classifier based on nearest shrunken centroids to identify a potential biomarker profile. We were able to identify 9 proteins that are needed to discriminate between good and poor responders shown in Table 3 and illustrated in Fig. 4.

**Table 3:** Classification to nearest shrunken centroids for identification of the most class-discriminating proteins

Acc. Nr.	Protein name	Gene name	log_2_ fold change (t-test difference) upregulated in poor responder	*p* value	t-test significant	Functional role
P21266	Glutathione S-transferase Mu 3	GSTM3	2.68	7.13E − 07	*	Uptake and detoxification of endogenous compounds and xenobiotics at the blood brain barrier [80]; many anticancer drugs are substrates for GST and, therefore, overexpression of GST is responsible for resistance to anti-cancer drugs in tumor cell lines [81]
P61962	DDB1- and CUL4-associated factor 7	DCAF7	2.06	1.11E − 07	*	Substrate receptor for a ubiquitin-protein ligase complex; involved in the pathway protein ubiquitination; involved in normal and disease skin development [82]; has been shown to function as a scaffold protein for protein complexes involved in kinase signalling [83]
P62937	Peptidyl-prolyl cis–trans isomerase A	PPIA	0.75	4.98E − 07		Upregulated in resistant human breast cancer cell line (vs. sensitive cell line) [84]; PPIases accelerate the folding of proteins; catalyzes the cis–trans isomerization of proline imidic peptide bonds in oligopeptides [85]
Q16864	V-type proton ATPase subunit F	ATP6V1F	3.12	5.47E − 07	*	V-ATPases are responsible for acidifying intracellular compartments [86]; an acidic environment leads to inactivation of T cells [56, 57]; supports the hypothesis that resistance is caused by the tumor inactivating immune cells
Q6FI81	Anamorsin	CIAPIN1	2.46	2.38E − 05		Anti-apoptotic effects in the cell; involved in negative control of cell death upon cytokine withdrawal [87]; may participate in breast cancer multi drug resistance (MDR) by regulating MDR1 and P53 expression, changing cell cycle and enhancing the anti-apoptotic capability of cells [88]
Q6UWP2	Dehydrogenase/reductase SDR family member 11	DHRS11	2.10	1.64E − 07	*	Involved in estrogen biosynthesis, which is part of steroid biosynthesis [89]; proposed role in sex hormone, neurosteroid, androgen, estrogen and bile acid metabolism; mRNA highly expressed in testis, small intestine, colon, kidney and cancer cell lines [90]
Q7Z7E8	Ubiquitin-conjugating enzyme E2 Q1	UBE2Q1	1.80	3.14E − 05		Catalyzes the covalent attachment of ubiquitin to other proteins [91]; may function as an oncogene that induces proliferation of cancer cells, and could be a novel diagnostic tool and a potential therapeutic target for colorectal cancer (CRC) [92]
Q8IVD9	NudC domain-containing protein 3	NUDCD3	2.42	2.78E − 05		Interacts selectively and non-covalently with an unfolded protein; functions to maintain the stability of dynein intermediate chain [93]; depletion of this gene product results in aggregation and degradation of dynein intermediate chain, mislocalization of the dynein complex from kinetochores, spindle microtubules, and spindle poles, and loss of gamma-tubulin from spindle poles [94]
Q8N4Q0	Prostaglandin reductase 3	ZADH2	2.48	2.44E − 06	*	Negatively modulates adipogenesis through regulation of PPARγ activity [95]; knockdown of prostaglandin reductase 1 (PTGR1) suppresses prostate cancer cell proliferation by inducing cell cycle arrest and apoptosis [96]

The table shows UniProt accession number (Acc. Nr.), protein name, gene name, t-test difference between LFQ values of poor and good responding patients (fold change, logarithmic scale to the base of two) with corresponding *p* values and t-test significance (rows with a t-test result above s0 = 0.5 and FDR 0.01 are reported as significant)

### EMT feature as a major classifier between good and poor prognosis validated in primary melanoma cell cultures

**Fig. 5:**
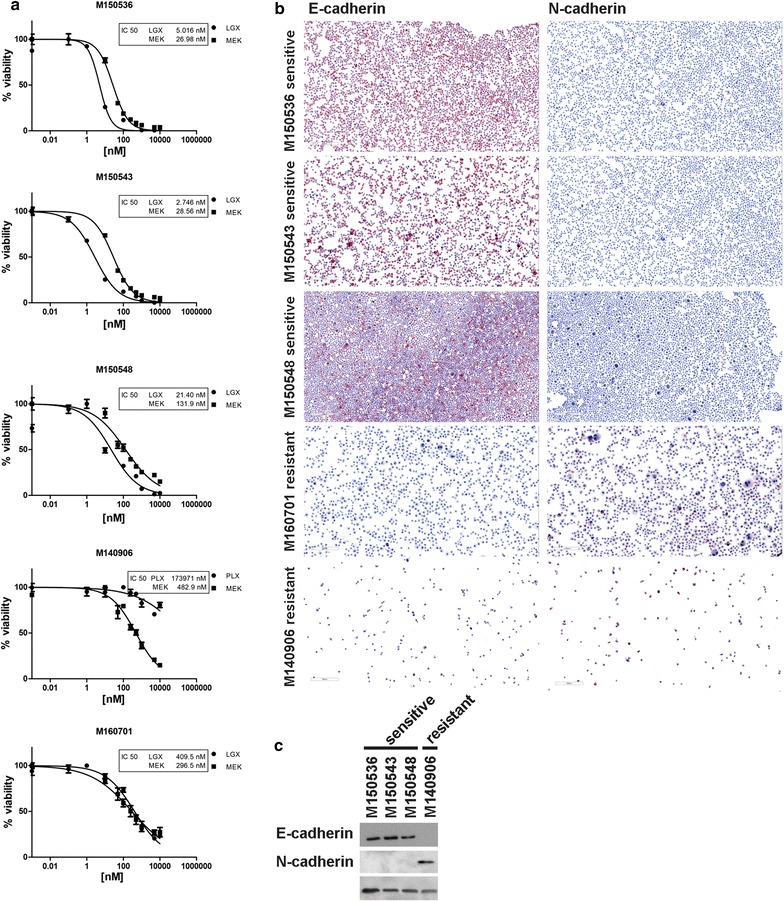
Primary melanoma cell cultures derived from cerebral metastases. Stratification of the samples by proliferation and viability assay and calculation of the IC50 for BRAF/MEK inhibitors (**a**). Sensitive cells show E-cadherin positivity and N-cadherin negativity in immunocytochemistry (**b**) and Western blot (**c**), whereas resistant cells show E-cadherin negativity and N-cadherin positivity

With the LC–MS shotgun approach we could show that samples from patients who responded poorly to MAPKi treatment showed a significantly higher expression of Cadherin-2 (CDH2), also known as N-Cadherin. For independent validation, we performed immunocytochemistry on primary melanoma cell cultures derived from cerebral melanoma metastases and Western blot on the lysates of these cells with E- and N-cadherin antibodies. The melanoma cells are classified as either sensitive (n = 3) or resistant (n = 2) based on the IC50 values for the BRAF/MEK inhibitors as determined by a viability assay. By these experiments we could show that the sensitive cells are E-cadherin positive, whereas the resistant cells were N-cadherin positive. This data strongly suggests EMT as a major classifier between good and poor prognosis (Fig. 5).

### Validation of discriminative signature by CPL/MUW proteome database

To further assess the discriminative signature (Table 3, Fig. 4) on protein level we tested the candidates using CPL/MUW proteome database [30], including 255 cell cultures, cell states and tissue, leading to a high protein similarity (57.14%) to the resistant melanoma cell line TMFI [30, 53]. In addition, we found one protein of our signature exclusively in TMFI, supporting our observation that this set of proteins is apparently characteristic for resistance (Additional file 5: Fig. S3).

### Validation and quantification of discriminative proteins by a targeted LC–MS analysis

**Fig. 6:**
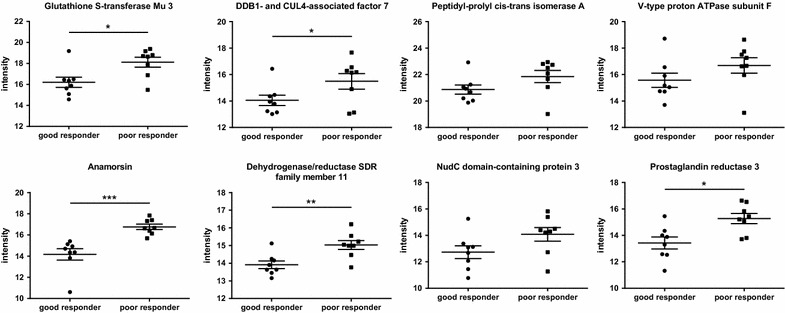
Intensities from the targeted MS approach for 8 of the 9 proteins from the discriminative signature listed in Table 3. Statistics for this plot were done in MSstats (*p value < 0.05, **p value < 0.01, ***p value < 0.001)

Furthermore, we validated and quantified the discriminative protein signature (Table 3, Fig. 4) with a targeted MS approach. We were able to validate our previous findings by the shotgun MS screen demonstrating significant protein expression between the groups of good and poor responding patients (Fig. 6).

### EMT-like signature induced by TGFβ can be correlated to the signature of poor responder

**Fig. 7:**
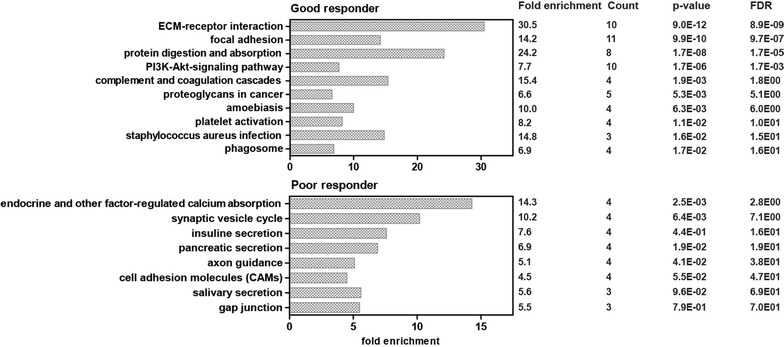
Functional annotation categories calculated in DAVID (DAVID Bioinformatics Resources 6.7, National Institute of Allergy and Infectious Diseases) of correlating genes upregulated by TGFβ (EMT genes) to proteins up-regulated in brain metastasis of melanoma patients treated with BRAF and MEK inhibitors (see Additional file 4: Table S2 for full list of overlapping data between TGFβ induced signature in Microarray data and shotgun proteomics data in cerebral melanoma metastases). Fold enrichment values, count (genes involved in the term), *p* value and FDR (false discovery rate, calculated using the Benjamini–Hochberg procedure), listed next to the graph, were calculated using DAVID bioinformatics resources

TGFβ-induced epithelial-to-mesenchymal-like transition (EMT-like) has been described in melanoma recently [25] and is characterized by melanoma cells switching from a proliferative phenotype to an invasive phenotype which is accompanied by induced drug-resistance to BRAF and MEK inhibitor treatment [24, 54]. Here we have correlated genes upregulated by TGFβ (EMT genes) to proteins up-regulated in brain metastasis of melanoma patients treated with BRAF and MEK inhibitors (see Fig. 7 for functional annotation categories (DAVID Bioinformatics Resources 6.7, National Institute of Allergy and Infectious Diseases) and Additional file 6: Table 3 for full list of overlapping data between TGFβ induced signature in microarray data and shotgun proteomics data in cerebral melanoma metastases). Correlation of factors regulated in TGFβ induced EMT with the proteome signature of the patients that showed poor response was significant.

### Correlation of the resistance signature to patient survival

**Fig. 8:**
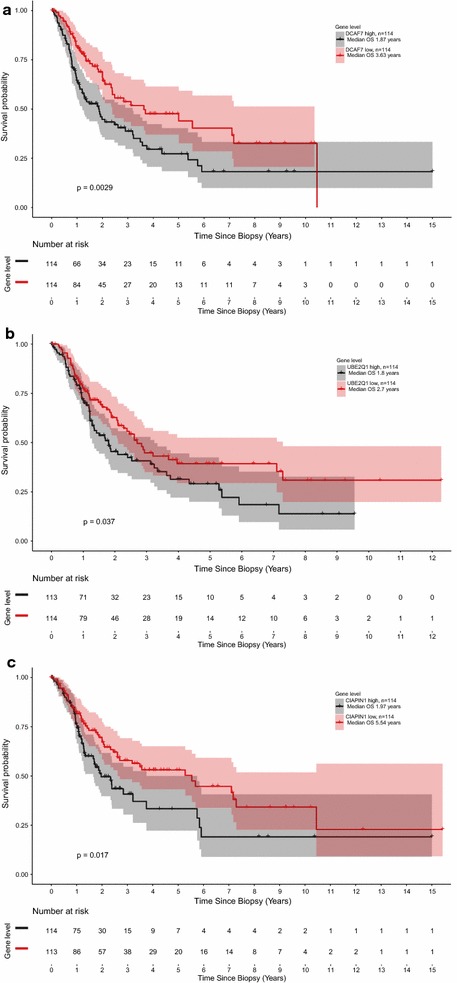
Kaplan-meier plots visualizing the survival of patients for DDB1- and CUL4-associated factor 7 (**a**), Ubiquitin-conjugating enzyme E2 Q1 (**b**) and Anamorsin (**c**) based on The Cancer Genome Atlas dataset (TCGA, cutaneous melanoma dataset (n = 456))

To demonstrate clinical relevance of the protein signature we researched the melanoma dataset (n = 456) of The Cancer Genome Atlas (TCGA) [51] and were able to show that three candidates, DDB1- and CUL4-associated factor 7, Ubiquitin-conjugating enzyme E2 Q1 and Anamorsin, out of the 9 discriminative proteins correlate with patients’ survival (Fig. 8).

### Increased immune cell presence in the cerebral metastases of good responder

**Fig. 9:**
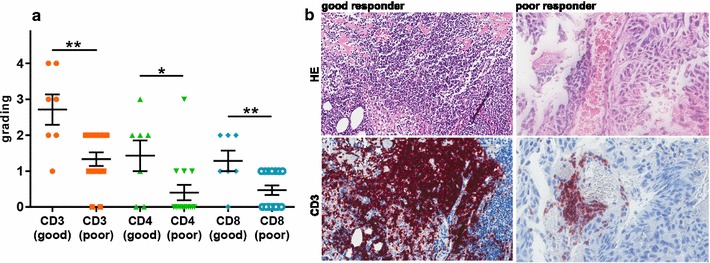
Immunohistochemistry of FFPE cerebral melanoma metastases cohort (n = 22). Evaluation of good responders (n = 7) versus poor responders (n = 15) shows significant different expression of he T cell marker CD3 (orange; *p* value = 0.002), CD4 (green; *p* value = 0.025) and CD8 (blue; *p* value = 0.007) visualized in the scatter plot (**a**) and by examples of the staining results (**b**)

The proteome signature of good responders was defined also by the up-regulation of immunogenic markers which we confirmed by immunohistochemical staining on the different T cell populations (CD3, CD4, CD8) on the FFPE cohort of clinical samples. These samples were classified as either good responder (n = 7) or poor responder (n = 15) based on PFS. The stained slides were evaluated by a dermatopathologist. Here we were able to demonstrate that poor response is characterized by less immune cell infiltrate in melanoma brain metastases, confirming our observation of higher immunological potential in the good responder (Fig. 9).

## Discussion

In this study design we used a five-step approach from MS proteome analysis up to clinical validation of patient samples in order to describe two distinct protein expression signatures defining treatment outcome to MAPK inhibition of patients with metastatic melanoma to the brain. Starting with shotgun MS to identify differentially expressed proteins between a set of good and poor responders with brain metastasis to targeted therapy. Next a variety of pathway analysis tools, GO terms, KEGG pathways, and GSEA, to define a set of biological and signaling pathways to distinguish between the good and poor responders. In step three we applied the nearest shrunken centroid classifier method and were able to define a 9 protein signature. Discriminative proteins were validated in step four by CPL/MUW proteome database and an EMT signature in vitro in melanoma cells derived from brain metastasis resistant to MAPK inhibition. Our next step was further validation and also quantification over a targeted MS approach. This was followed by a functional validation of the EMT signature. Finally, in the last step we also showed clinical validation by correlating survival of patients with the resistant signature and performed immunohistochemistry on cerebral melanoma metastases. Employing proteomic analysis, we confirmed known extra-cerebral resistance mechanisms in cerebral metastases and further uncovered possible brain specific mechanisms of drug efflux, which might serve as treatment targets or as predictive markers for these kinds of metastases.

After our initial screen using shotgun LC–MS, we investigated pathways differing most apparently between good and poor responders. The MAPK signaling pathway was most reassuring since it represents one of the main resistance mechanisms known in melanoma [55].

We then searched for protein candidates, which played a role in these pathways and performed literature search in order to investigate whether these markers play a role in resistance mechanisms (Fig. 3).

V-type proteins, a group of proton ATPases, are significantly up-regulated in poor responders. V-type proton ATPases transport hydrogen ions (H+) across membranes, which may cause an acidic environment and thus lead to inactivation of T cells [56, 57]. This might support the hypothesis that resistance is supported by inactivating immune cells. V-type proteins might also be involved in drug efflux; their role in acidifying the ECM and thus maintaining multi-drug-resistance (MDR) properties has already been shown [58].

Eukaryotic translation initiation factors (EIFs) are pivotal in cancer progression and involved in different hallmarks of cancer [59, 60]. Complexes of this group of proteins have been shown to function as a nexus of resistance to anti-BRAF and anti-MEK cancer therapies [61] and were also found up-regulated in the samples of patients with poor response to MAPKi treatment.

The overrepresentation of a large group of neuronal proteins (Table 1) matches with their suspected role in cell migration and metastasis. Here, where the movement of cells is controlled, similar processes as in axon guiding are happening. A group of secreted axon guidance molecules, involved in neuronal development, has already been discussed and proven to be overexpressed in various carcinomas, as well as melanoma. It was also shown that the expression was correlated with advanced stage and grade of cancer [62]. Since the involvement of neuronal axon guiding factors in metastasis has been demonstrated by previous studies [63], it can be hypothesized, that a reinforced neuronal profile may be a sign of increased migration and resistance to MAPKi of brain metastases, making neuronal proteins a potential target for future therapeutic approaches.

Demonstrated by the heterogeneity in therapeutic resistance, the plasticity of melanoma cells might allow the tumor to adapt to biological processes, for example by EMT-like mechanisms. There are various specific molecular events associated with EMT, such as increased production of ECM components, activation of transcription factors and reorganization and expression of cytoskeletal proteins [21, 28]. EMT is also associated with induction of resistance and metastasis [26, 28, 29]. Especially, the down-regulation of E-cadherin is balanced by the increased expression of mesenchymal neural cadherin (N-cadherin). This, so called cadherin switch, alters cell adhesion [26, 27] and is considered to be a fundamental event in EMT, leading to a loss of cell–cell contact, increased invasive properties and typical morphological changes [21, 28, 29]. EMT is associated with an induction of the mesenchymal marker N-cadherin or Cadherin-2 (CDH2), which we have shown to be significantly up-regulated in poor responders. The biological reason behind the changes to a mesenchymal phenotype is the increased capability to detach from the epithelial layer and to gain the ability for migration [28].

The concept of phenotype switching, first described in melanoma by Hoek et al. [19, 20], characterizes two transcriptional distinct melanoma cell populations, a proliferative and an invasive type. The phenotype-switching model accounts for disease progression and tumor heterogeneity, as well as aspects of resistance [22]. Melanoma cells are able to switch back and forth between these two phenotypes [21]. Also, melanoma cells have shown to change their phenotype from proliferative to invasive state in response to targeted therapy with BRAFi and MEKi, which is associated with drug resistance. Furthermore, proliferative melanoma cells have been shown to be more responsive to MAPK pathway inhibition than invasive phenotype cells, independently of their mutation status [23, 24]. The major role in phenotype switching and the transition from a proliferative to an invasive cell type and ultimately leading to metastasis is related to EMT-like mechanisms [25]. Identifying critical switches in EMT processes and finding a way to block these, might serve as a strategy to prevent metastasis and to decrease therapeutic resistance. The presently characterized mechanisms might thus not only provide insight into mechanisms of resistance but may also increase the understanding of the metastasis processes. In this pilot study, we were able to detect EMT features, as well as an increase of proteins involved in calcium ion binding and cell adhesion in line with previous published data [53]. Interaction with the ECM is also important for metastasis, since cells need to be able to adhere. A recently published study also exhibited a higher capability of cell adherence demonstrating that Vemurafenib resistant cells undergoing EMT have an increased ability to interact with ECM proteins [64].

Possible potential lies in the identified groups of exchanger and transporter proteins (Table 1), which we detected an over-representation in the poor responder patient group. There are candidates among the transporter proteins, which are not only involved in leukocyte migration but also in many transport processes. It can therefore be hypothesized that these transport activities also take place in drug efflux, leading or contributing to therapy resistance. Enhanced drug efflux membrane transporters has already been described in chemoresistance [65]. Na+/H+ exchangers or antiporters have been associated with cancer metastasis and invasion [66–69], making them valuable for future drug development [70].

Patients who responded well to MAPKi treatment revealed a huge number of significantly up-regulated immune response related proteins such as myeloperoxidase (MPO), HLA proteins and immunoglobulin chains. Although these patients were not treated with immunotherapy, the immune system is always involved in cancer defense. This leads to the presumption that in patients who showed good response to therapy, melanoma is better recognizable for the immune system via these up-regulated receptors and fragments. It also has recently been shown that many subtypes of HLA molecules are down-regulated during MAPKi resistance [71], supporting the good response of patients with up-regulated HLA proteins. MPO is a peroxidase enzyme expressed in professional phagocytic cells, most abundantly in neutrophils. It is involved in cellular homeostasis and of particular interest because of its important role in the initiation and progression of acute and chronic inflammatory diseases [72]. Although in some cases of neoplastic malignancies there is research pointing out the actively tumor promoting function of immune inflammatory cells [60], there are also mechanisms known by which adaptive immune cells modulate cancer for example by cytokine-mediated lysis of tumor cells [73]. ECM structural constituents, as well as proteins involved in cell adhesion were significantly up-regulated amongst patients who showed a good response to therapy. Compared to poor responders, in this case the cell adhesion molecules are involved in immune response, leukocyte migration and ECM organization. This accounts for the activation of the immune system and may be due to the organism attacking the metastases. Apolipoproteins might be another interesting group for further investigation. In patients with good treatment response they were significantly up-regulated and have already been described as a probable prognostic factor and can be predictive for survival [74].

Finally, we wanted to be able to differentiate between good and poor responding patients and translate our data towards clinical application. To identify a potential biomarker profile, we constructed a classifier based on nearest shrunken centroids. We identified 9 proteins that are necessary to discriminate between good and poor responding patients, namely Glutathione S-transferase Mu 3, DDB1- and CUL4-associated factor 7, Peptidyl-prolyl cis–trans isomerase A, Anamorsin, Dehydrogenase/reductase SDR family member 11, Ubiquitin-conjugating enzyme E2 Q1, NudC domain-containing protein 3, Prostaglandin reductase 3 and V-type proton ATPase subunit F (Fig. 4). Literature research demonstrated that these candidates are involved in resistance or tumor progression and exert novelty in the field of melanoma resistance (Table 3).

With the targeted approach we were successfully able to validate 8 out of the 9 proteins.

We also made use of the TCGA database, which provides clinical information and gene expression of 456 melanoma samples. Here we were able to correlate DDB1- and CUL4-associated factor 7 (DCAF7), Ubiquitin-conjugating enzyme E2 Q1 (UBE2Q1) and Anamorsin (CIAPIN1) also on gene expression level with poor survival, demonstrating clinical relevance of the protein signature.

As previously described EMT plays a crucial role in metastasis. By inducing EMT in a cell culture melanoma model with previous sensitivity, we detected the typical signature also identified in the poor responding patients. This fortifies EMT as a major classifier between good and poor prognosis. We could validate the observation that EMT is an important process in resistance in brain metastases of melanoma by staining on the main factors involved in EMT (E- and N-cadherin) by immunocytochemistry and Western blotting.

High amounts of tumor-infiltrating lymphocytes (TILs), high ratios of PD-1+/CD8+ cells and high levels of PD-L1 were shown to be negatively correlated with brain metastases size, although there was no significant association of patient survival with TILs [75]. Another study showed that T-cell specific genes such as CD3 and CD8 are expressed at higher levels in patients with a prolonged overall survival [76]. Meaning any molecule associated with the presence of T cells implies a better outcome, which is consistent with the published literature about the association of TILs with good prognosis. Concerning TILs, CD3+ and CD8+ T cells may be the most important effector population during regression of melanoma metastasis [77]. T cell infiltrated melanomas, especially those with high CD8+ cells, are more likely to be associated with PD-L1 expression in tumor cells, an increased prognosis and increased time to develop brain metastasis [78]. With our FFPE cohort we were able to demonstrate that poor response is characterized by a lower infiltrate of immune cells in melanoma brain metastases confirming our observation of the over-representation of immunogenic marker in the good responder. This is in line with recent literature and gives new insights into MAPKi resistance and brain metastases. This leads us to the assumption that also MAPKi resistant patients could benefit from immunotherapy-induced activation of their immune system, since we see higher immune infiltration in tumors of patients that responded well to therapy.

## Conclusions

Drug resistance mechanisms are content of latest research. Calcium ion binding proteins, EIFs, EMT, lysosomal pathway and others have already been identified by previous proteome analyses as main features associated with resistance. With our tissue proteomic approach, we were able to detect similar resistance mechanisms in cerebral metastases, indicating resemblance to visceral metastases. The cerebral metastases of poorly responding patients expressed transporter and exchanger proteins in a higher amount, which might, especially in the brain, be responsible for drug efflux and thus contribute to therapy resistance. With patients who showed good response to therapy the immunogenic signature indicates a better response to targeted therapy combined with immunotherapy. This suggests that patients with a poor response to MAPKi might also benefit from activating their immune system. Further studies analyzing the function of these targets are warranted. Evaluation of new treatment options in vitro is necessary to verify possible candidates. Additional studies with increased sample size will also give more profound insights into the pathomechanisms of MAPKi resistance. Intensive research is demanded for the detection of promising therapeutics and to understand resistance mechanisms. Primary tumors collected today are from huge relevance, not only for research but also for the patient. The availability of adequate sample material for future studies will determine the validity of the gained evidence. By initiating appropriate biobank approaches, future scientific endeavors to develop novel therapies or stratification of patients for better treatment can be ensured [79].

## Additional files

**Additional file 1: Figure S1.** Study outline shows the experimental design of the study.

**Additional file 2: Table S1.** Clinical table with age, gender, treatment, response distribution, PFS, mutational status, all number of samples, IC50 of the cell systems and the applied methods.

**Additional file 3: Figure S2.** Complement and coagulation cascades (A) are enriched in good responder. Cell adhesion molecules (B), calcium signaling pathway (C) and MAPK signaling pathway (D) are highly enriched and up-regulated in poor responder, as visualized by GSEA.

**Additional file 4: Table S2.** Statistical output for GSEA (gene set enrichment analysis). Nominal p-value, false discovery rate (FDR) and familywise-error rate (FWER) are given for the for significantly enriched protein sets.

**Additional file 5: Figure S3.** Correlation of proteins (P62937, P61962, Q6FI81, P21266) identified by nearest shrunken centroid and CPL/MUW proteome database, including 255 cell cultures, cell states and tissue, leading to a high protein similarity to the resistant melanoma cell line TMFI.

**Additional file 6: Table S3.** Genes upregulated in melanoma cells by TGFβ signaling correlating with proteins that were also found to be upregulated in the cerebral melanoma metastases by proteomics.

## Abbreviations

ACN: acetonitrile
Acc. Nr.: accession number
BP: biological process
BSA: bovine serum albumin
CC: cellular component
DTT: dithiothreitol
ECM: extracellular matrix
EIF: eukaryotic translation initiation factor
EMT: epithelial to mesenchymal transition
FA: formic acid
FBS: fetal bovine serum
FDR: false discovery rate
FFPE: formalin-fixed paraffin-embedded
GO: gene ontology
GSEA: gene set enrichment analysis
HCD: higher-energy collisional dissociation
HLA: human leukocyte antigen
IAA: iodoacetamide
KEGG: Kyoto Encyclopedia of Genes and Genomes
LFQ: label-free quantification
Ig: immunoglobulin
MAPK: mitogen-activated protein kinase
MEK: mitogen-activated protein kinase
MF: molecular function
MPO: myeloperoxidase
MWCO: molecular weight cut-off
PBS: phosphate buffered saline
PFS: progression-free survival
ppm: parts per million
SDS-PAGE: sodium dodecyl sulfate polyacrylamide gel electrophoresis
TCGA: The Cancer Genome Atlas
TFA: trifluoroacetic acid
TILs: tumor-infiltrating lymphocytes
