# Supplementary material for: Proteomics-based insights into mitogen-activated protein kinase inhibitor resistance of cerebral melanoma metastases
Source: Clin Proteomics. 2018 Mar 9;15:13. doi: 10.1186/s12014-018-9189-x (PMC5844114; doi:10.1186/s12014-018-9189-x)
Supplement: Supplementary file 1 — Additional file 1: Figure S1. Study outline shows the experimental design of the study. [file 12014_2018_9189_MOESM1_ESM.pdf]

Screening by Shotgun MS

Tissue samples of cerebral melanoma metastases: poor responder n=5, good responder n=13

A) **EMT** by DAVID, KEGG and GSEA for poor responder

B) **9 proteins signature** by nearest shrunken centroid for poor responder

C) **Immune activity** for good responder

Validation

1. **Primary melanoma cell cultures** defined by IC50 to MAPKi resistant n=2, sensitive n=3

1.1 **ICC Staining** *correlation for A*

1.2 **WB** *correlation for A*

2. Correlation of 9 proteins signature (correlation for *B*) to resistance by **CPL/MUW proteome database**

Quantification and Validation

Targeted MS approach

1. **Tissue samples of shotgun MS**
   poor responder n=7, good responder n=4
2. **added Tissue samples**
   poor responder n=1, good responder n=4

correlation for B

Clinical relevance

1. **TGCA** melanoma samples
   n=456, *correlation for B*
2. **IHC**
   poor responder n=6 (samples: 15), good responder n=5 (samples: 7)
   correlation for C

Functional verification

- EMT induction by TGFβ
- Identification of signature of poor responder
