# Supplementary material for: Proteomics-based insights into mitogen-activated protein kinase inhibitor resistance of cerebral melanoma metastases
Source: Clin Proteomics. 2018 Mar 9;15:13. doi: 10.1186/s12014-018-9189-x (PMC5844114; doi:10.1186/s12014-018-9189-x)

Supplementary figure 2: Complement and coagulation cascades (A) are enriched in good responder. Cell adhesion molecules (B), calcium signaling pathway (C) and MAPK signaling pathway (D) are highly enriched and up-regulated in poor responder, as visualized by GSEA.

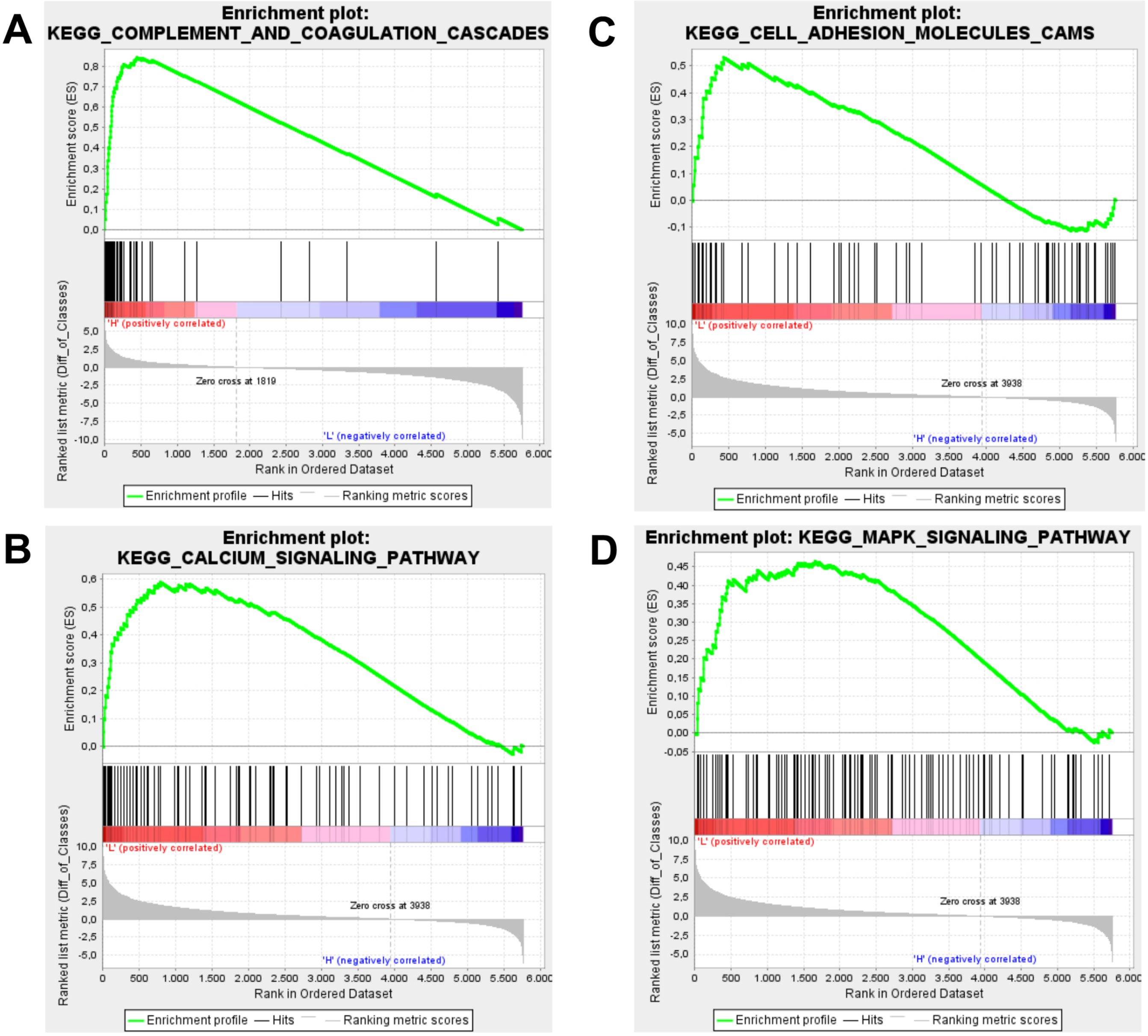

Supplement: Supplementary file 3 — Additional file 3: Figure S2. Complement and coagulation cascades (A) are enriched in good responder. Cell adhesion molecules (B), calcium signaling pathway (C) and MAPK signaling pathway (D) are highly enriched and up-regulated in poor responder, as visualized by GSEA. [file 12014_2018_9189_MOESM3_ESM.pdf]
