# Supplementary material for: Proteomics-based insights into mitogen-activated protein kinase inhibitor resistance of cerebral melanoma metastases
Source: Clin Proteomics. 2018 Mar 9;15:13. doi: 10.1186/s12014-018-9189-x (PMC5844114; doi:10.1186/s12014-018-9189-x)
Supplement: Supplementary file 4 — Additional file 4: Table S2. Statistical output for GSEA (gene set enrichment analysis). Nominal p-value, false discovery rate (FDR) and familywise-error rate (FWER) are given for the for significantly enriched protein sets. [file 12014_2018_9189_MOESM4_ESM.docx]

Supplementary table 2: Statistical output for GSEA (gene set enrichment analysis).

| Name | NOM p-value | FDR q-value | FWER p-value |
| --- | --- | --- | --- |
| KEGG_CELL_ADHESION_MOLECULES_CAMS | 0.009100101 | 0.11631556 | 0.987 |
| KEGG_CALCIUM_SIGNALING_PATHWAY | 0.0010121458 | 0.029044397 | 0.295 |
| KEGG_MAPK_SIGNALING_PATHWAY | 0.03722334 | 0.2764605 | 1.0 |
| KEGG_COMPLEMENT_AND_COAGULATION_CASCADES | 0.0 | 0.0 | 0.0 |

Nominal p-value, false discovery rate (FDR) and familywise-error rate (FWER) are given for the for significantly enriched protein sets.
