# Supplementary material for: Proteomics-based insights into mitogen-activated protein kinase inhibitor resistance of cerebral melanoma metastases
Source: Clin Proteomics. 2018 Mar 9;15:13. doi: 10.1186/s12014-018-9189-x (PMC5844114; doi:10.1186/s12014-018-9189-x)

Supplementary figure 3: Correlation of proteins (P62937, P61962, Q6FI81, P21266) identified by nearest shrunken centroid and CPL/MUW protein database, including 255 cell cultures, cell states and tissue, leading to a high protein similarity to the resistant melanoma cell line TMFI.

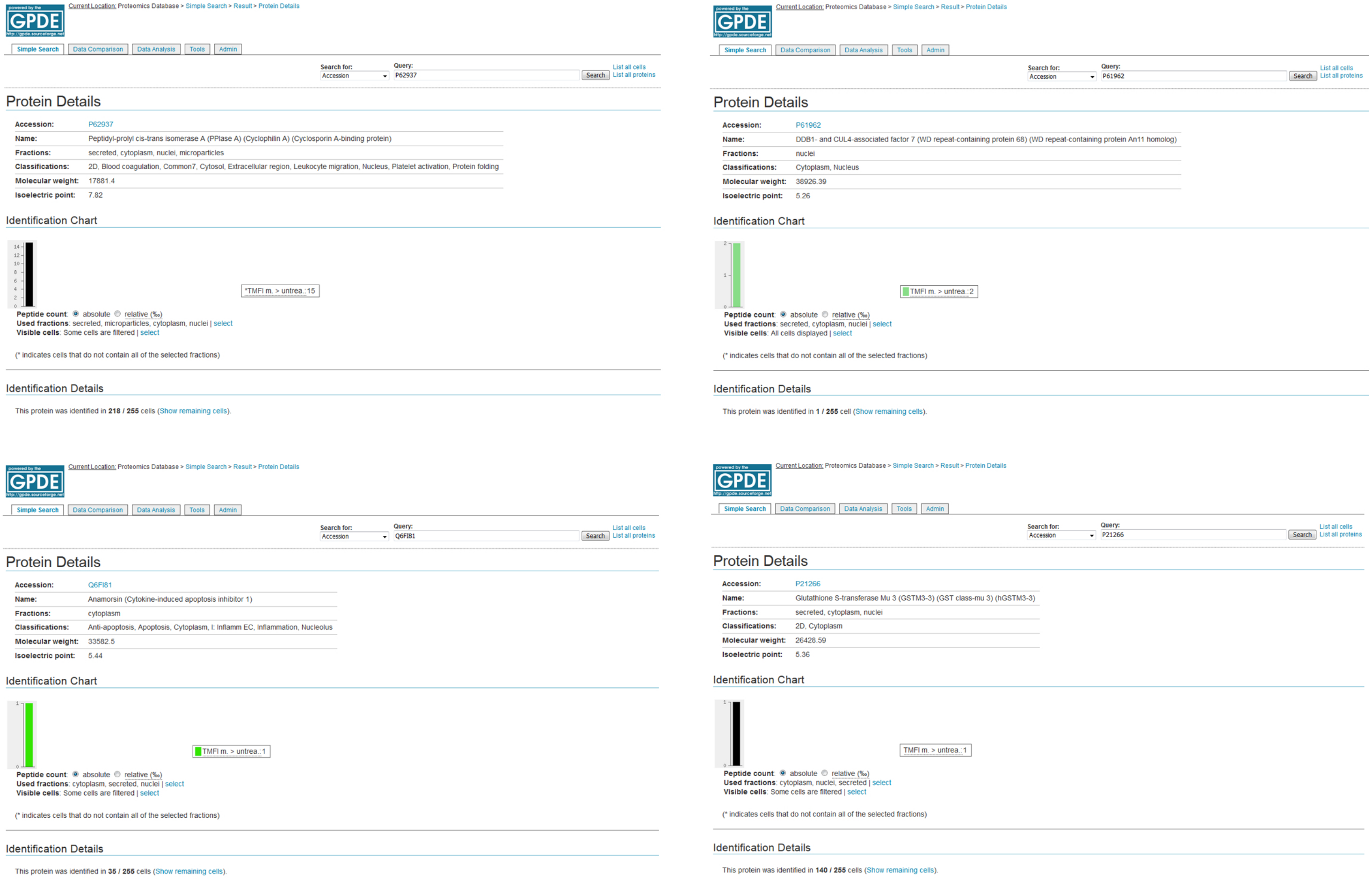

Supplement: Supplementary file 5 — Additional file 5: Figure S3. Correlation of proteins (P62937, P61962, Q6FI81, P21266) identified by nearest shrunken centroid and CPL/MUW proteome database, including 255 cell cultures, cell states and tissue, leading to a high protein similarity to the resistant melanoma cell line TMFI. [file 12014_2018_9189_MOESM5_ESM.pdf]
