# Supplementary material for: Proteomics-based insights into mitogen-activated protein kinase inhibitor resistance of cerebral melanoma metastases
Source: Clin Proteomics. 2018 Mar 9;15:13. doi: 10.1186/s12014-018-9189-x (PMC5844114; doi:10.1186/s12014-018-9189-x)
Supplement: Supplementary file 6 — Additional file 6: Table S3. Genes upregulated in melanoma cells by TGFβ signaling correlating with proteins that were also found to be upregulated in the cerebral melanoma metastases by proteomics. [file 12014_2018_9189_MOESM6_ESM.docx]

Supplementary table 3: Genes upregulated in melanoma cells by TGF$\beta$ signaling correlating with proteins that were also found to be upregulated in the cerebral melanoma metastases by proteomics.

| Gene names | Fold change (by RNAseq) | Accession | Protein names | log_2_ fold change (t-test difference; by proteomics) | p-value | Upregulated in (by proteomics) |
| --- | --- | --- | --- | --- | --- | --- |
| LUM | 3,0975 | P51884 | Lumican | 4,60337 | 0,000890806 | good responder |
| COL1A1 | 145,8623482 | P02452 | Collagen alpha-1(I) chain | 3,66635 | 0,0217585 | good responder |
| COL1A2 | 21,03386809 | P08123 | Collagen alpha-2(I) chain | 3,23554 | 0,0125135 | good responder |
| FBN1 | 29,30211082 | P35555 | Fibrillin-1 | 3,22959 | 0,106788 | good responder |
| S100A8 | 2,938496583 | P05109 | Protein S100-A8 | 3,17054 | 0,000210573 | good responder |
| ITGB3 | 2,644773358 | P05106 | Integrin beta-3 | 3,0503 | 0,000272144 | good responder |
| LTBP2 | 3,778637771 | Q14767 | Latent-transforming growth factor beta-binding protein 2 | 3,04873 | 0,000545489 | good responder |
| PRELP | 2,779338843 | P51888 | Prolargin | 2,85921 | 0,073466 | good responder |
| COL6A2 | 3,525 | P12110 | Collagen alpha-2(VI) chain | 2,77952 | 0,0950356 | good responder |
| FN1 | 2,232869081 | P02751 | Fibronectin | 2,61116 | 0,004261 | good responder |
| C1S | 2,608036391 | P09871 | Complement C1s subcomponent | 2,50711 | 0,000812013 | good responder |
| TGFBI | 5,009575923 | Q15582 | Transforming growth factor-beta-induced protein ig-h3 | 2,38412 | 0,102768 | good responder |
| S100A4 | 2,304788214 | P26447 | Protein S100-A4 | 2,31658 | 0,0339069 | good responder |
| COL6A3 | 11,02777778 | P12111 | Collagen alpha-3(VI) chain | 2,28247 | 0,0683806 | good responder |
| SFRP1 | 2,645248869 | Q8N474 | Secreted frizzled-related protein 1 | 2,03644 | 0,000136259 | good responder |
| CFH | 2,08750835 | P08603 | Complement factor H | 1,93553 | 0,000307742 | good responder |
| MYOF | 3,336775082 | Q9NZM1 | Myoferlin | 1,85407 | 0,0701808 | good responder |
| HLA-B | 2,146596859 | Q95365 | HLA class I histocompatibility antigen, B-38 alpha chain | 1,84147 | 0,00363696 | good responder |
| COL12A1 | 2,832692308 | Q99715 | Collagen alpha-1(XII) chain | 1,75031 | 0,291012 | good responder |
| BPGM | 2,42251462 | P07738 | Bisphosphoglycerate mutase | 1,73029 | 0,0024661 | good responder |
| MFGE8 | 2,175378267 | Q08431 | Lactadherin | 1,70766 | 0,0446549 | good responder |
| CLU | 11,15698587 | P10909 | Clusterin | 1,65115 | 0,00137754 | good responder |
| COL6A1 | 3,637396694 | P12109 | Collagen alpha-1(VI) chain | 1,64905 | 0,149561 | good responder |
| AEBP1 | 7,3421875 | Q8IUX7 | Adipocyte enhancer-binding protein 1 | 1,57756 | 0,0271124 | good responder |
| GGT2 | 2,114443567 | P36268 | Inactive gamma-glutamyltranspeptidase 2 | 1,54354 | 0,0230096 | good responder |
| A2M | 19,18803419 | P01023 | Alpha-2-macroglobulin | 1,51006 | 0,000241679 | good responder |
| COL15A1 | 6,048599671 | P39059 | Collagen alpha-1(XV) chain | 1,48231 | 0,0446469 | good responder |
| MFAP2 | 2,301324503 | P55001 | Microfibrillar-associated protein 2 | 1,41722 | 0,088687 | good responder |
| ALOX5 | 5,167976424 | P09917 | Arachidonate 5-lipoxygenase | 1,4038 | 0,00520825 | good responder |
| CFI | 3,689066059 | P05156 | Complement factor I | 1,35934 | 0,00995438 | good responder |
| ABI3BP | 2,357783211 | Q7Z7G0 | Target of Nesh-SH3 | 1,32148 | 0,0429 | good responder |
| TMEM2 | 4,59571323 | Q9UHN6 | Transmembrane protein 2 | 1,29663 | 0,212848 | good responder |
| IGFBP7 | 2,592712551 | Q16270 | Insulin-like growth factor-binding protein 7 | 1,2496 | 0,21024 | good responder |
| MYH9 | 2,124178232 | P35579 | Myosin-9 | 1,24254 | 0,00182142 | good responder |
| FLNA | 2,4375 | P21333 | Filamin-A | 1,20416 | 0,0444684 | good responder |
| TCHH | 2,445187854 | Q07283 | Trichohyalin | 1,18654 | 0,0356628 | good responder |
| COL5A2 | 2,396472393 | P05997 | Collagen alpha-2(V) chain | 1,17124 | 0,18572 | good responder |
| VASN | 3,715867159 | Q6EMK4 | Vasorin | 1,15757 | 0,0742233 | good responder |
| LTBP1 | 2,677655678 | Q14766 | Latent-transforming growth factor beta-binding protein 1 | 1,15488 | 0,0460518 | good responder |
| AQP1 | 9,31 | P29972 | Aquaporin-1 | 1,149 | 0,0676434 | good responder |
| THBS2 | 2,987447699 | P35442 | Thrombospondin-2 | 1,13988 | 0,238895 | good responder |
| ITGB5 | 6,905008636 | P18084 | Integrin beta-5 | 1,10813 | 0,162371 | good responder |
| ITIH5 | 2,303251493 | Q86UX2 | Inter-alpha-trypsin inhibitor heavy chain H5 | 1,10586 | 0,0815351 | good responder |
| TF | 6,366471735 | P02787 | Serotransferrin | 1,05722 | 0,00388726 | good responder |
| SNAP25 | 3,9 | P60880 | Synaptosomal-associated protein 25 | 4,45644 | 0,000601942 | poor responder |
| L1CAM | 2,411965812 | P32004 | Neural cell adhesion molecule L1 | 4,26902 | 1,91E-06 | poor responder |
| SYNGR3 | 2,128912467 | O43761 | Synaptogyrin-3 | 4,24321 | 0,000360928 | poor responder |
| PCSK1N | 2,311577312 | Q9UHG2 | ProSAAS | 3,91446 | 0,000421924 | poor responder |
| NEGR1 | 2,314166231 | Q7Z3B1 | Neuronal growth regulator 1 | 3,7139 | 0,00192917 | poor responder |
| NCS1 | 2,172011662 | P62166 | Neuronal calcium sensor 1 | 3,55111 | 6,65E-05 | poor responder |
| VAMP1 | 2,868536372 | P23763 | Vesicle-associated membrane protein 1 | 3,48154 | 0,00175263 | poor responder |
| ATP1B1 | 3,165 | P05026 | Sodium/potassium-transporting ATPase subunit beta-1 | 3,28499 | 0,00236684 | poor responder |
| NRN1 | 2,769170579 | Q9NPD7 | Neuritin | 3,23701 | 0,000742892 | poor responder |
| NTM | 10,20448179 | Q9P121 | Neurotrimin | 3,12849 | 0,00444966 | poor responder |
| SYT7 | 2,71557377 | O43581 | Synaptotagmin-7 | 3,09894 | 0,00165938 | poor responder |
| AK5 | 2,795135641 | Q9Y6K8 | Adenylate kinase isoenzyme 5 | 3,02576 | 0,000154238 | poor responder |
| GJA1 | 4,979426892 | P17302 | Gap junction alpha-1 protein | 2,71644 | 0,0102183 | poor responder |
| TSC22D1 | 2,007194245 | Q15714 | TSC22 domain family protein 1 | 2,66974 | 1,65E-06 | poor responder |
| BASP1 | 31,1092952 | P80723 | Brain acid soluble protein 1 | 2,66216 | 0,0135805 | poor responder |
| KIFAP3 | 2,15432526 | Q92845 | Kinesin-associated protein 3 | 2,60381 | 5,52E-05 | poor responder |
| OXCT1 | 3,307159353 | P55809 | Succinyl-CoA:3-ketoacid coenzyme A transferase 1, mitochondrial | 2,59349 | 0,000129641 | poor responder |
| BAIAP2 | 2,409474367 | Q9UQB8 | Brain-specific angiogenesis inhibitor 1-associated protein 2 | 2,5744 | 9,31E-06 | poor responder |
| DNM1 | 4,056932966 | Q05193 | Dynamin-1 | 2,55651 | 0,000473552 | poor responder |
| TANC2 | 2,369127517 | Q9HCD6 | Protein TANC2 | 2,54931 | 3,62E-05 | poor responder |
| CDH2 | 3,428482199 | P19022 | Cadherin-2 | 2,52857 | 0,00102269 | poor responder |
| CALB2 | 4,550669216 | P22676 | Calretinin | 2,45179 | 0,00228248 | poor responder |
| CLSTN1 | 2,036814425 | O94985 | Calsyntenin-1 | 2,41912 | 0,00697458 | poor responder |
| ASPA | 2,748951782 | P45381 | Aspartoacylase | 2,39345 | 0,000783118 | poor responder |
| S100A1 | 2,674708819 | P23297 | Protein S100-A1 | 2,39048 | 0,00393515 | poor responder |
| CPE | 4,09942005 | P16870 | Carboxypeptidase E | 2,35795 | 0,0177293 | poor responder |
| TUBB2B | 3,025862069 | Q9BVA1 | Tubulin beta-2B chain | 2,32598 | 0,00464602 | poor responder |
| ROGDI | 2,002199413 | Q9GZN7 | Protein rogdi homolog | 2,27567 | 0,00045292 | poor responder |
| DNM3 | 11,19675926 | Q9UQ16 | Dynamin-3 | 2,2721 | 0,00441814 | poor responder |
| MARCKS | 2,060820368 | P29966 | Myristoylated alanine-rich C-kinase substrate | 2,27139 | 0,00470807 | poor responder |
| PIGT | 2,057532173 | Q969N2 | GPI transamidase component PIG-T | 2,21616 | 0,00110232 | poor responder |
| NOVA2 | 4,596103896 | Q9UNW9 | RNA-binding protein Nova-2 | 2,18684 | 0,0016991 | poor responder |
| CADM1 | 3,995221843 | Q9BY67 | Cell adhesion molecule 1 | -2,15926 | 0,0709895 | poor responder |
| DPYSL2 | 2,008928571 | Q16555 | Dihydropyrimidinase-related protein 2 | 2,10298 | 0,00491425 | poor responder |
| KCNMA1 | 15,84745763 | Q12791 | Calcium-activated potassium channel subunit alpha-1 | 2,06321 | 0,000329954 | poor responder |
| PRKAR2B | 2,529 | P31323 | cAMP-dependent protein kinase type II-beta regulatory subunit | 2,03004 | 0,00160512 | poor responder |
| SCG5 | 12,4045 | P05408 | Neuroendocrine protein 7B2 | 1,97332 | 0,00512066 | poor responder |
| PLXNA4 | 3,554 | Q9HCM2 | Plexin-A4 | 1,95771 | 0,0128106 | poor responder |
| CD59 | 2,01728024 | P13987 | CD59 glycoprotein | 1,95552 | 0,0861224 | poor responder |
| MPI | 2,030683403 | P34949 | Mannose-6-phosphate isomerase | 1,91021 | 0,0132375 | poor responder |
| HPCAL1 | 2,380246914 | P37235 | Hippocalcin-like protein 1 | 1,90962 | 5,74E-05 | poor responder |
| SCG2 | 8,142163662 | P13521 | Secretogranin-2 | 1,89199 | 0,0582476 | poor responder |
| PGM2L1 | 3,951142132 | Q6PCE3 | Glucose 1,6-bisphosphate synthase | 1,88168 | 0,00928763 | poor responder |
| PTGFRN | 2,223642173 | Q9P2B2 | Prostaglandin F2 receptor negative regulator | 1,80262 | 0,000216328 | poor responder |
| DPYSL4 | 2,700642792 | O14531 | Dihydropyrimidinase-related protein 4 | 1,80124 | 0,0198433 | poor responder |
| MRAS | 2,532616487 | O14807 | Ras-related protein M-Ras | 1,75944 | 0,00788388 | poor responder |
| SPP1 | 62,95007564 | P10451 | Osteopontin | 1,75645 | 0,0149819 | poor responder |
| CADM4 | 3,225806452 | Q8NFZ8 | Cell adhesion molecule 4 | 1,74339 | 0,0253155 | poor responder |
| GNB5 | 2,066995769 | O14775 | Guanine nucleotide-binding protein subunit beta-5 | 1,72344 | 0,059934 | poor responder |
| S100A13 | 2,826446281 | Q99584 | Protein S100-A13 | 1,71176 | 0,000406549 | poor responder |
| MOG | 8,861683849 | Q16653 | Myelin-oligodendrocyte glycoprotein | 1,70951 | 0,291338 | poor responder |
| SYNPO | 2,330442324 | Q8N3V7 | Synaptopodin | 1,68707 | 0,0178253 | poor responder |
| PLXND1 | 2,311048557 | Q9Y4D7 | Plexin-D1 | 1,6631 | 0,0160698 | poor responder |
| STK38L | 2,026449644 | Q9Y2H1 | Serine/threonine-protein kinase 38-like | 1,64143 | 0,0294146 | poor responder |
| ALDOC | 2,055555556 | P09972 | Fructose-bisphosphate aldolase C | 1,63941 | 0,00729763 | poor responder |
| NES | 2,121212121 | P48681 | Nestin | 1,63724 | 0,0900537 | poor responder |
| NISCH | 2,673659674 | Q9Y2I1 | Nischarin | 1,63506 | 0,00966115 | poor responder |
| KALRN | 2,874224978 | O60229 | Kalirin | 1,5756 | 0,0805992 | poor responder |
| TLN2 | 3,902646503 | Q9Y4G6 | Talin-2 | 1,54086 | 0,000818466 | poor responder |
| EPHA4 | 3,774216524 | P54764 | Ephrin type-A receptor 4 | 1,53193 | 0,0763211 | poor responder |
| FAM162A | 2,843629344 | Q96A26 | Protein FAM162A | 1,5142 | 0,0045712 | poor responder |
| ENO2 | 3,221183801 | P09104 | Gamma-enolase | 1,49683 | 0,0373489 | poor responder |
| GLB1 | 2,000720981 | P16278 | Beta-galactosidase | 1,47339 | 0,103679 | poor responder |
| TRIO | 2,044579534 | O75962 | Triple functional domain protein | 1,47025 | 0,00598948 | poor responder |
| ITFG1 | 2,190561529 | Q8TB96 | T-cell immunomodulatory protein | 1,46834 | 0,0215428 | poor responder |
| NBEA | 2,585627938 | Q8NFP9 | Neurobeachin | 1,44093 | 0,0351515 | poor responder |
| GAA | 2,19711903 | P10253 | Lysosomal alpha-glucosidase | 1,43871 | 0,051574 | poor responder |
| TMED4 | 2,100286533 | Q7Z7H5 | Transmembrane emp24 domain-containing protein 4 | 1,41956 | 0,071824 | poor responder |
| ATP6V1G2 | 2,018976423 | O95670 | V-type proton ATPase subunit G 2 | 1,39594 | 0,000410638 | poor responder |
| CTSB | 4,88951049 | P07858 | Cathepsin B | 1,38648 | 0,107021 | poor responder |
| PRKCA | 3,794634598 | P17252 | Protein kinase C alpha type | 1,32799 | 0,0215553 | poor responder |
| SORBS1 | 2,493559719 | Q9BX66 | Sorbin and SH3 domain-containing protein 1 | 1,32581 | 0,00306269 | poor responder |
| NPTXR | 2,547833935 | O95502 | Neuronal pentraxin receptor | 1,30356 | 0,101733 | poor responder |
| SH3BGRL3 | 2,404163053 | Q9H299 | SH3 domain-binding glutamic acid-rich-like protein 3 | 1,29556 | 0,017341 | poor responder |
| GOPC | 2,2595 | Q9HD26 | Golgi-associated PDZ and coiled-coil motif-containing protein | 1,28884 | 0,0010806 | poor responder |
| PTPRS | 3,181525242 | Q13332 | Receptor-type tyrosine-protein phosphatase S | 1,27869 | 0,0912172 | poor responder |
| RASA1 | 2,051 | P20936 | Ras GTPase-activating protein 1 | 1,21969 | 0,0106704 | poor responder |
| P4HA1 | 2,96984127 | P13674 | Prolyl 4-hydroxylase subunit alpha-1 | 1,21729 | 0,165299 | poor responder |
| ASMTL | 3,243406179 | O95671 | N-acetylserotonin O-methyltransferase-like protein | 1,19635 | 0,0160648 | poor responder |
| CLIP2 | 2,607970343 | Q9UDT6 | CAP-Gly domain-containing linker protein 2 | 1,16825 | 0,019352 | poor responder |
| MMP14 | 2,607361963 | P50281 | Matrix metalloproteinase-14 | 1,16729 | 0,0312429 | poor responder |
| RAB27B | 5,38375 | O00194 | Ras-related protein Rab-27B | 1,1474 | 0,0700068 | poor responder |
| NDRG1 | 5,155446756 | Q92597 | Protein NDRG1 | 1,14059 | 0,0823492 | poor responder |
| MACROD2 | 2,719617225 | A1Z1Q3 | O-acetyl-ADP-ribose deacetylase MACROD2 | 1,13949 | 0,033459 | poor responder |
| PAM | 2,363244176 | P19021 | Peptidyl-glycine alpha-amidating monooxygenase | 1,13922 | 0,0333036 | poor responder |
| LGI4 | 2,298679868 | Q8N135 | Leucine-rich repeat LGI family member 4 | 1,12965 | 0,0743686 | poor responder |
| APLP1 | 2,527350427 | P51693 | Amyloid-like protein 1 | 1,11759 | 0,0399326 | poor responder |
| TAX1BP3 | 2,466981132 | O14907 | Tax1-binding protein 3 | 1,11458 | 0,0543314 | poor responder |
| FSCN1 | 2,328703704 | Q16658 | Fascin | 1,10891 | 0,000412385 | poor responder |
| P4HA2 | 4,355281207 | O15460 | Prolyl 4-hydroxylase subunit alpha-2 | 1,04951 | 0,12159 | poor responder |
| APLP2 | 2,554711246 | Q06481 | Amyloid-like protein 2 | 1,04635 | 0,07502 | poor responder |
| UBE2I | 2,181089744 | P63279 | SUMO-conjugating enzyme UBC9 | 1,02818 | 0,0632045 | poor responder |
